# Supplementary material for: An updated C. elegans nuclear body muscle transcriptome for studies in muscle formation and function
Source: Skelet Muscle. 2023 Mar 2;13:4. doi: 10.1186/s13395-023-00314-2 (PMC9979539; doi:10.1186/s13395-023-00314-2)
Supplement: Supplementary file 1 — Additional file 1: Fig. S1. Nuclei stability overtime. Fig. S2. The C. elegans body muscle dataset. Fig. S3. Muscle fiber diagram showing the main components of the sarcomere. Fig. S4. Promoter region analysis for genes detected in this study. Fig. S5. RT qPCR approach to validate body muscle miRNAs identified from nuclear sequencing. Fig. S6. miRNA validation using a modified RT-qPCR approach. [file 13395_2023_314_MOESM1_ESM.pdf]

Additional File 1 for:

An updated *C. elegans* body muscle transcriptome for studies in muscle formation and function

Anna L Schorr, Alejandro Felix Mejia, Martina Y Miranda and Marco Mangone

* To whom correspondence should be addressed. mangone@asu.eu

This PDF includes:

Figs. S1-S6

Table of Contents

Additional File 1

Fig. S1: Nuclei stability overtime ……..……………………..………………………………..3

Fig. S2: The *C. elegans* body muscle dataset ....…………........….4

Fig. S3: Muscle fiber diagram showing the main components of the sarcomere 5

Fig. S4: Promoter region analysis for genes detected in this study ..…………............6

Fig. S5: : RT qPCR approach to validate body muscle miRNAs identified from nuclear sequencing ..……………………………………………………………............7

Fig. S6: miRNA validation using a modified RT-qPCR approach..……….....................8

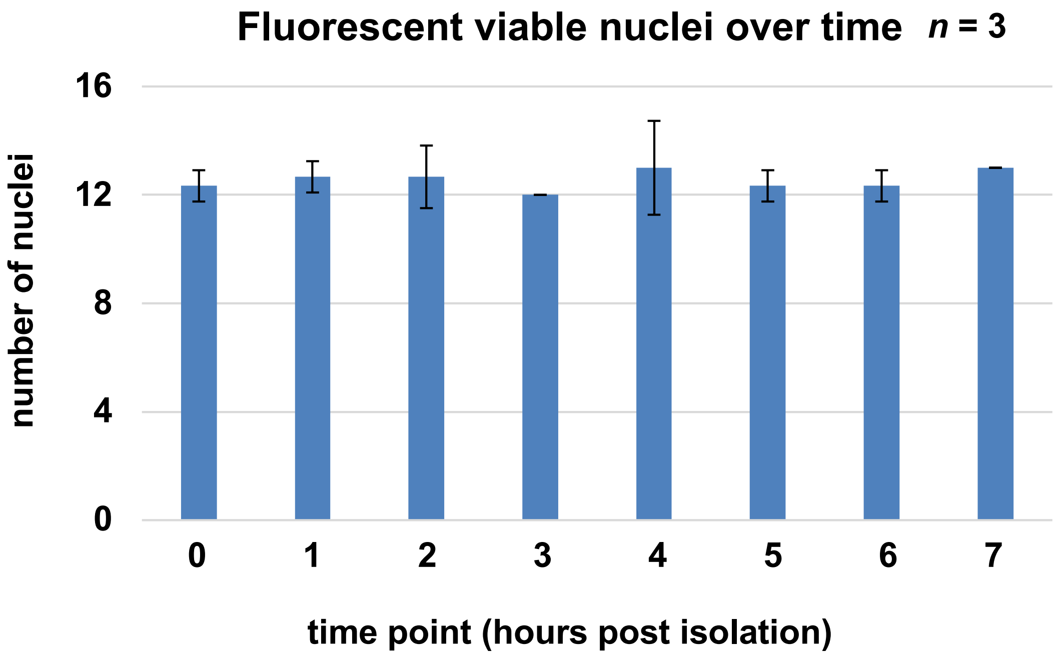

Supplemental Figure S1: nuclei stability overtime: The numbers of fluorescent, viable nuclei were recorded over eight time periods: time 1 as the start time, or time 0 after nuclei isolation (pre-FACS) and each time point afterwards equal to one hour on the X axis. The recorded number of mCherry fluorescent nuclei are indicated on the Y axis. Processing of the images and calculations of number of nuclei was done through imageJ software. Samples were imaged in triplicate.

A

samples		reads total	reads mapped	reads mapped (%)
**set1**	sample	61,094,976	59,521,548	97.42
	replicate	52,785,598	51,118,381	96.84
	**total**	**113,880,574**	**110,639,939**	**97.15**
**set2**	sample	47,432,446	46,155,817	97.31
	replicate	51198034	49,722,949	97.12
	**total**	**98,630,480**	**95,878,666**	**97.21**
**BN452 nuclear**	rep1	43,941697	36,768,398	83.48
	rep2	31,100,828	26,239,776	84.37

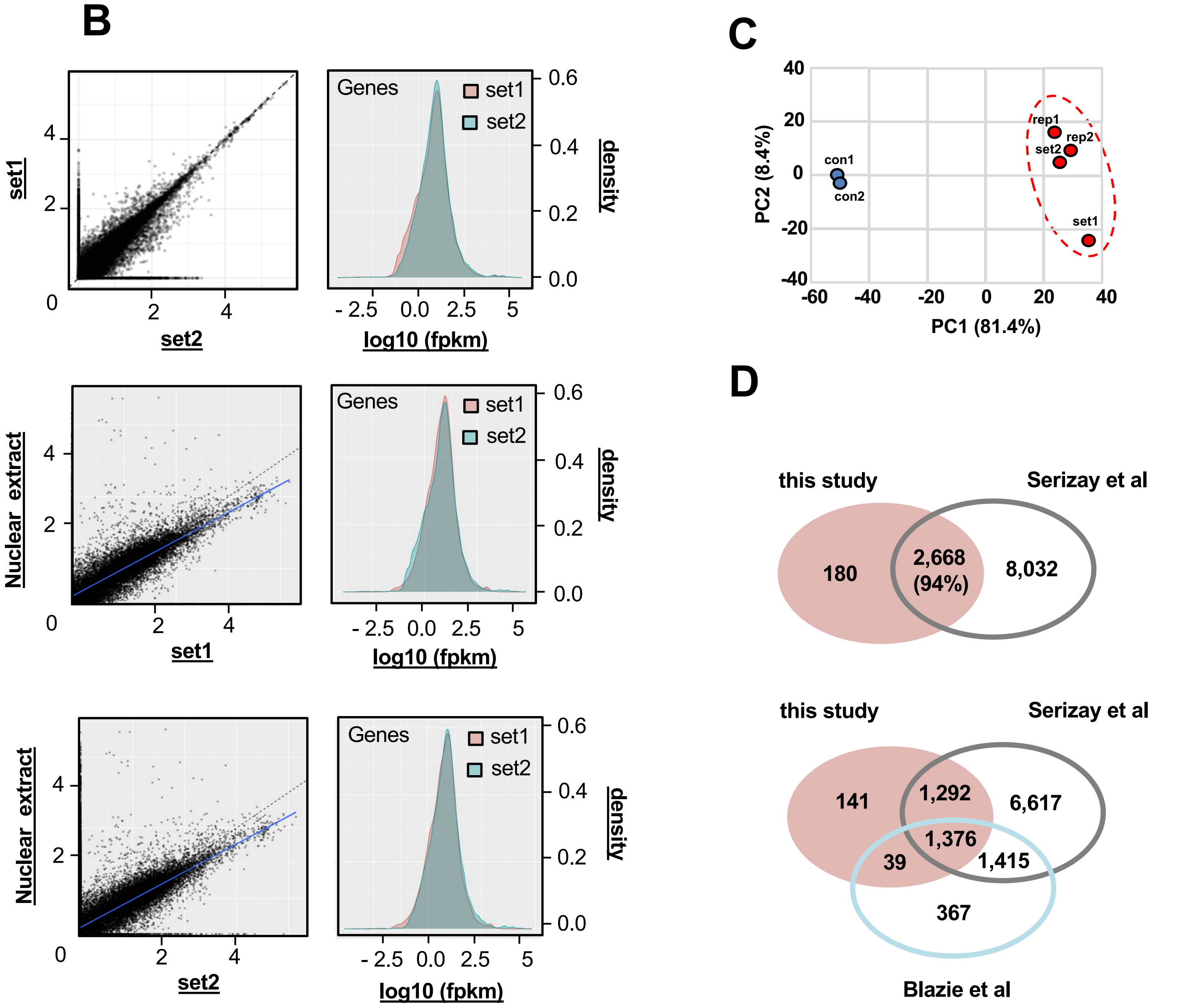

Supplemental Figure S2: The *C. elegans* muscle Dataset. (A) Sequencing Summary. Set1 and set2: Pooled datasets from each experimental sample and its technical replicate. 'BN452 nuclear: Pooled group containing RNA extracted from the BN452 strain (two technical replicates). (B) The distribution of the *fpkm* values in the experiment (blue) and replicate (orange) samples for each dataset. The plots were generated using the cummeRbund package v. 2.0. (C) Principal Component Analysis (PCA) shows a high correlation among each duplicate within our datasets. (D) Body muscle Venn diagram comparisons between this study (mixed stages; *fpkm*>=5; pink), Serizay et al., (L4 Larvae; TPM>=5; grey), and Blazie et al. (mixed stages; *fpkm>=1*; blue).

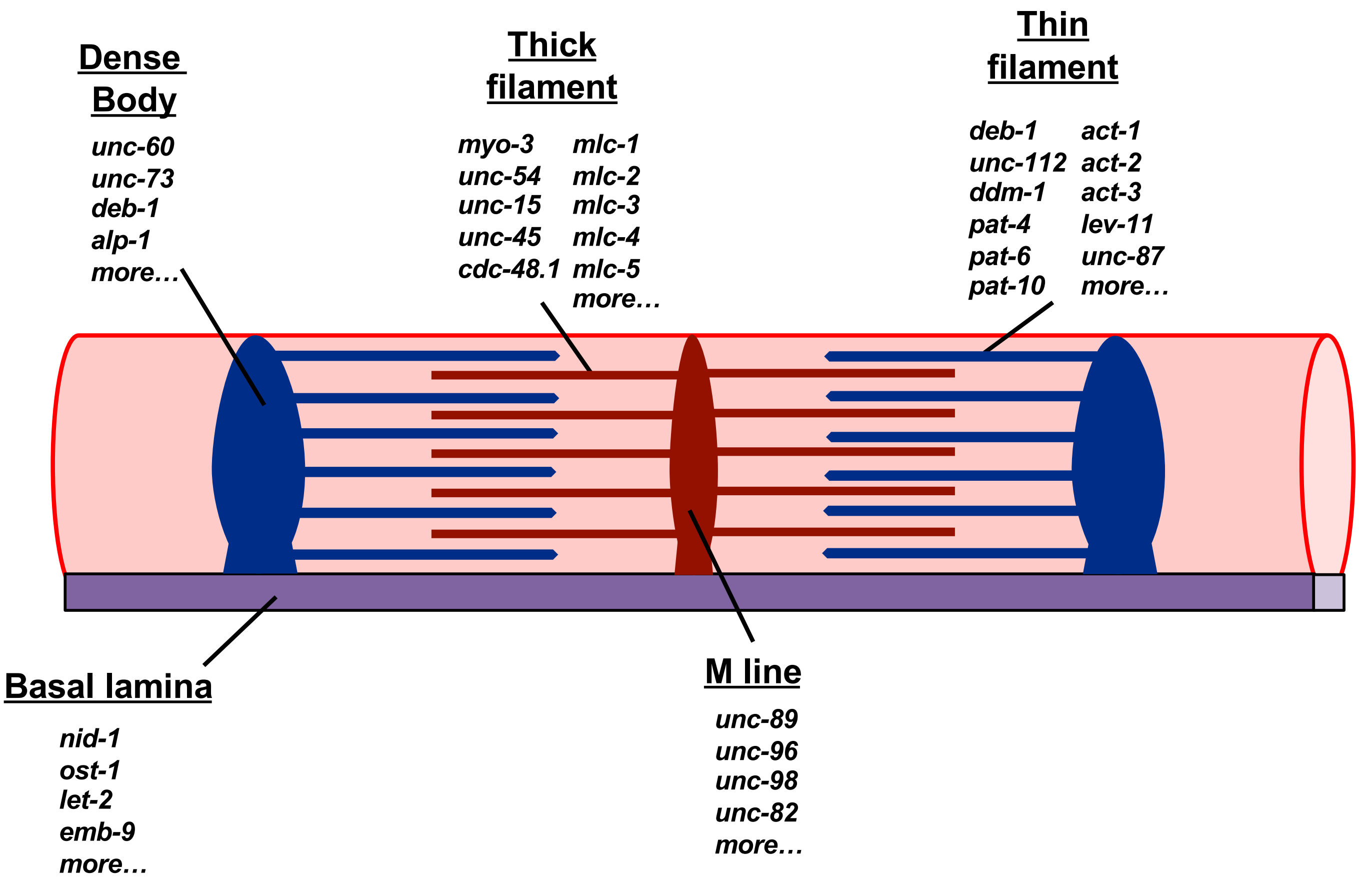

Supplemental Figure S3: Muscle fiber diagram showing the main components of the sarcomere. The names of the main structures are underscored. The genes shown below each structure were identified in this study and have been previously assigned as members of this structure.

A

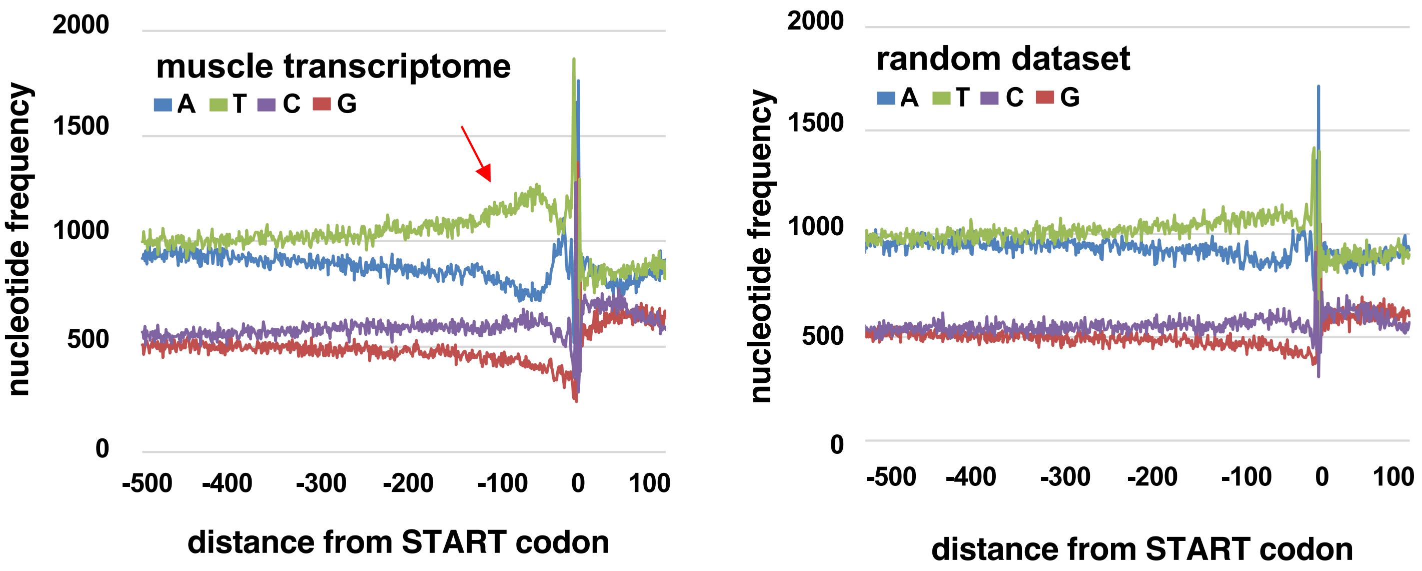

B

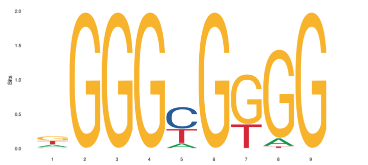

KLF1

P-value	E-value	Q-value	Enrichment	Threshold
9.51e-34	1.97e-30	7.77e-32	2.44	9.09

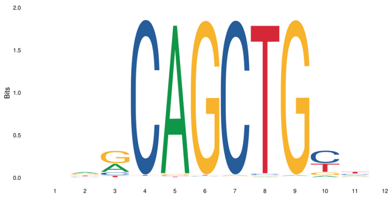

MYOG

P-value	E-value	Q-value	Enrichment	Threshold
4.21e-18	8.70e-15	8.34e-17	1.41	6.32

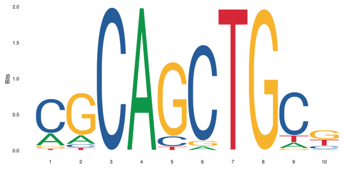

NHLH1

P-value	E-value	Q-value	Enrichment	Threshold
5.37e-9	1.11e-5	3.36e-8	1.54	8.80

Figure S4: Promoter region analysis for genes detected in this study. We extracted and studied the DNA regions 500bp upstream from the start codon for each of 2,848 genes unique in our muscle dataset. (A) The average base composition for promoter regions in all muscle transcripts (top), and a random dataset of the same number of genes (bottom). We detected a strong enrichment of thymidine within 100nts upstream of the transcription start site (red arrow). (B) Motif analysis. Three enriched promoter sequences are shown.

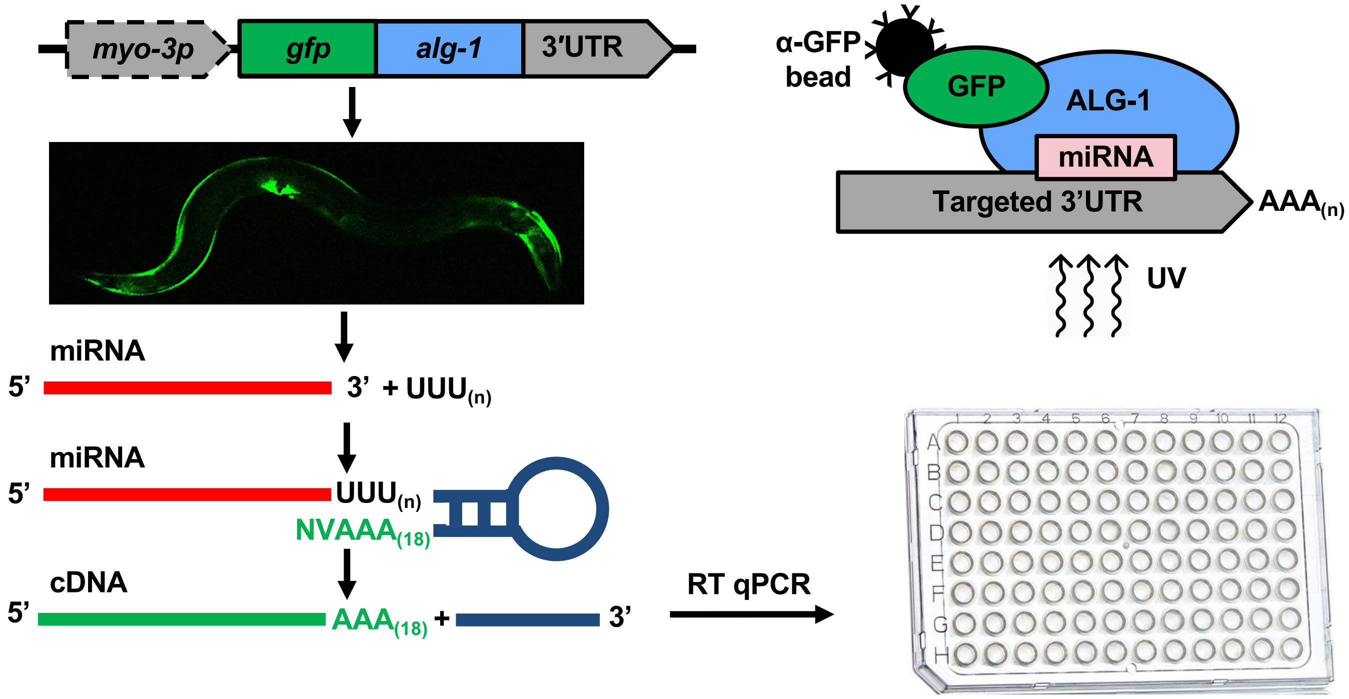

Figure S5: RT qPCR approach to validate body muscle miRNAs identified from nuclear sequencing. We adapted a previously described RT qPCR protocol for quantifying miRNAs. RNA was isolated from an immunoprecipitation of GFP::ALG-1 from transgenic worm strain *myo-3p::GFP::ALG-1* [23-33]. RNA was polyuridylated before performing the reverse transcriptase step using a stem-loop poly-A reverse primer containing a site for a universal reverse primer. MiRNA-specific forward primers for 5p and 3p isoforms were used to determine which strand is loaded into the Argonaute protein ALG-1.

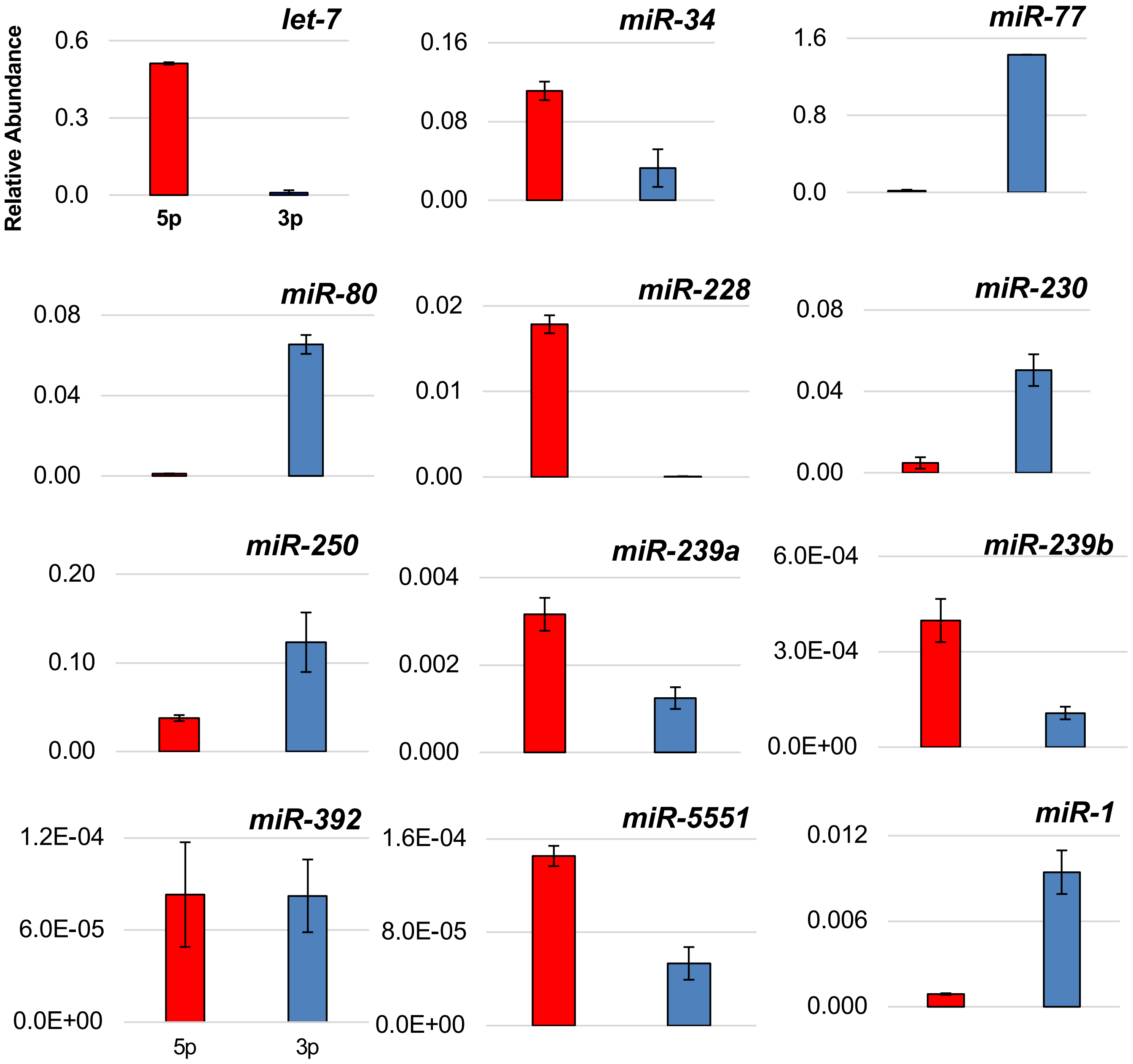

Figure S6: miRNA validation using a modified RT-qPCR approach. RT qPCRs were done in triplicate with forward primers specific 5p or 3p strands from miRNAs identified from body muscle tissue to demonstrate which strand is preferred for the Argonaute complex to incorporate as a mature miRNA. The relative abundance for each pair of miRNAs is shown on the Y-axis.
