# Supplementary material for: An updated C. elegans nuclear body muscle transcriptome for studies in muscle formation and function
Source: Skelet Muscle. 2023 Mar 2;13:4. doi: 10.1186/s13395-023-00314-2 (PMC9979539; doi:10.1186/s13395-023-00314-2)
Supplement: Supplementary file 3 — Additional file 3: Supplemental Table S2. Simple Enrichment Analysis of sequence motifs identified in promoters of transcripts identified in this study. [file 13395_2023_314_MOESM3_ESM.pdf]

SEA
Simple Enrichment Analysis

For further information on how to interpret these results please access https://meme-suite.org/meme/doc/sea-output-format.html.
To get a copy of the MEME software please access https://meme-suite.org.

If you use SEA in your research, please cite the following paper:
Timothy L. Bailey and Charles E. Grant, "SEA: Simple Enrichment Analysis of motifs", *BioRxiv*, 2021.

Enriched Motifs | Input Files | Program information | Results in TSV Format | Matching Sequences

Enriched Motifs

Found 387 motifs with *E*-values ≤ 10.

Logo	Database	ID	Alt ID	*P*-value	*E*-value	*Q*-value	TP	FP	Enrichment Ratio	Score Threshold	Positional Distribution	Matches per Sequence
	JASPAR2022 CORE vertebrates non-redundant	MA1723.1	MA1723.1.PRDM9	4.01e-58	4.91e-55	3.05e-55	1659 / 2695 (61.6%)	1072 / 2695 (39.8%)	1.55	3.40		
	JASPAR2022 CORE vertebrates non-redundant	MA0757.1	MA0757.1.ONECUT3	4.18e-55	5.11e-52	1.59e-52	442 / 2695 (16.4%)	106 / 2695 (3.9%)	4.14	12.00		
	JASPAR2022 CORE vertebrates non-redundant	MA1721.1	MA1721.1.ZNF93	9.82e-53	1.20e-49	2.48e-50	1299 / 2695 (48.2%)	757 / 2695 (28.1%)	1.72	4.02		
	JASPAR2022 CORE vertebrates non-redundant	MA0080.6	MA0080.6.Spi1	6.72e-43	8.23e-40	1.28e-40	989 / 2695 (36.7%)	538 / 2695 (20.0%)	1.84	8.56		
	JASPAR2022 CORE vertebrates non-redundant	MA0516.3	MA0516.3.SP2	8.59e-43	1.05e-39	1.30e-40	644 / 2695 (23.9%)	271 / 2695 (10.1%)	2.37	8.97		
	JASPAR2022 CORE vertebrates non-redundant	MA0742.2	MA0742.2.KLF12	6.04e-41	7.39e-38	7.64e-39	501 / 2695 (18.6%)	180 / 2695 (6.7%)	2.77	9.29		
	JASPAR2022 CORE vertebrates non-redundant	MA2025.1	MA2025.1.CTCF	8.90e-40	1.09e-36	9.65e-38	1686 / 2695 (62.6%)	1205 / 2695 (44.7%)	1.40	1.60		
	JASPAR2022 CORE vertebrates non-redundant	MA1630.2	MA1630.2.ZNF281	3.45e-38	4.22e-35	3.27e-36	985 / 2695 (36.5%)	559 / 2695 (20.7%)	1.76	1.89		
	JASPAR2022 CORE vertebrates non-redundant	MA1564.1	MA1564.1.SP9	8.82e-36	1.08e-32	7.44e-34	642 / 2695 (23.8%)	298 / 2695 (11.1%)	2.15	10.09		
	JASPAR2022 CORE vertebrates non-redundant	MA0746.2	MA0746.2.SP3	3.84e-35	4.70e-32	2.91e-33	607 / 2695 (22.5%)	275 / 2695 (10.2%)	2.20	7.99		
	JASPAR2022 CORE vertebrates non-redundant	MA0599.1	MA0599.1.KLF5	2.95e-34	3.61e-31	2.03e-32	595 / 2695 (22.1%)	270 / 2695 (10.0%)	2.20	9.74		
	JASPAR2022 CORE vertebrates non-redundant	MA0493.2	MA0493.2.KLF1	9.51e-34	1.16e-30	6.02e-32	499 / 2695 (18.5%)	204 / 2695 (7.6%)	2.44	9.09		
	JASPAR2022 CORE vertebrates non-redundant	MA1107.2	MA1107.2.KLF9	3.09e-33	3.78e-30	1.80e-31	567 / 2695 (21.0%)	254 / 2695 (9.4%)	2.23	9.16		
	JASPAR2022 CORE vertebrates non-redundant	MA1870.1	MA1870.1.KLF7	4.73e-31	5.79e-28	2.57e-29	481 / 2695 (17.8%)	202 / 2695 (7.5%)	2.37	10.77		
	JASPAR2022 CORE vertebrates non-redundant	MA1875.1	MA1875.1.ZNF669	5.68e-31	6.95e-28	2.87e-29	1735 / 2695 (64.4%)	1316 / 2695 (48.8%)	1.32	2.20		
	uniprobe mouse	UP00002_1	Sp4_primary	1.96e-30	2.40e-27	9.30e-29	583 / 2695 (21.6%)	278 / 2695 (10.3%)	2.09	9.10		
	JASPAR2022 CORE vertebrates non-redundant	MA0740.2	MA0740.2.KLF14	3.57e-30	4.38e-27	1.60e-28	486 / 2695 (18.0%)	209 / 2695 (7.8%)	2.32	9.90		
	uniprobe mouse	UP00093_1	Klf7_primary	7.23e-30	8.85e-27	3.05e-28	816 / 2695 (30.3%)	464 / 2695 (17.2%)	1.76	8.02		
	JASPAR2022 CORE vertebrates non-redundant	MA1713.1	MA1713.1.ZNF610	1.17e-29	1.43e-26	4.66e-28	2128 / 2695 (79.0%)	1758 / 2695 (65.2%)	1.21	1.30		
	JASPAR2022 CORE vertebrates non-redundant	MA0039.4	MA0039.4.KLF4	2.58e-29	3.16e-26	9.80e-28	726 / 2695 (26.9%)	394 / 2695 (14.6%)	1.84	9.65		
	JASPAR2022 CORE vertebrates non-redundant	MA1873.1	MA1873.1.ZNF530	3.31e-29	4.05e-26	1.20e-27	1470 / 2695 (54.5%)	1061 / 2695 (39.4%)	1.39	1.25		
	JASPAR2022 CORE vertebrates non-redundant	MA0081.2	MA0081.2.SPIB	3.93e-29	4.81e-26	1.36e-27	1096 / 2695 (40.7%)	710 / 2695 (26.3%)	1.54	7.72		
	JASPAR2022 CORE vertebrates non-redundant	MA1876.1	MA1876.1.ZNF701	2.13e-28	2.60e-25	7.01e-27	2335 / 2695 (86.6%)	2018 / 2695 (74.9%)	1.16	0.50		
	JASPAR2022 CORE vertebrates non-redundant	MA1511.2	MA1511.2.KLF10	1.32e-27	1.61e-24	4.16e-26	441 / 2695 (16.4%)	188 / 2695 (7.0%)	2.34	10.14		
	JASPAR2022 CORE vertebrates non-redundant	MA0685.2	MA0685.2.SP4	2.88e-27	3.53e-24	8.75e-26	613 / 2695 (22.7%)	316 / 2695 (11.7%)	1.94	6.22		
	JASPAR2022 CORE vertebrates non-redundant	MA0079.5	MA0079.5.SP1	5.43e-27	6.65e-24	1.59e-25	1008 / 2695 (37.4%)	646 / 2695 (24.0%)	1.56	4.68		
	uniprobe mouse	UP00099_1	Ascl2_primary	8.37e-27	1.02e-23	2.35e-25	2170 / 2695 (80.5%)	1828 / 2695 (67.8%)	1.19	4.18		
	uniprobe mouse	UP00021_1	Zfp281_primary	5.36e-26	6.56e-23	1.45e-24	1231 / 2695 (45.7%)	856 / 2695 (31.8%)	1.44	5.27		
	JASPAR2022 CORE vertebrates non-redundant	MA1866.1	MA1866.1.PATZ1	5.56e-26	6.80e-23	1.45e-24	1751 / 2695 (65.0%)	1371 / 2695 (50.9%)	1.28	0.01		
	JASPAR2022 CORE vertebrates non-redundant	MA0162.4	MA0162.4.EGR1	5.89e-26	7.21e-23	1.49e-24	590 / 2695 (21.9%)	305 / 2695 (11.3%)	1.93	9.72		
	JASPAR2022 CORE vertebrates non-redundant	MA1653.1	MA1653.1.ZNF148	8.46e-26	1.03e-22	2.07e-24	1291 / 2695 (47.9%)	914 / 2695 (33.9%)	1.41	5.82		
	JASPAR2022 CORE vertebrates non-redundant	MA1627.1	MA1627.1.Wt1	1.20e-25	1.47e-22	2.85e-24	1646 / 2695 (61.1%)	1265 / 2695 (46.9%)	1.30	4.97		
	uniprobe mouse	UP00080_1	Gata5_primary	1.50e-24	1.83e-21	3.45e-23	610 / 2695 (22.6%)	328 / 2695 (12.2%)	1.86	10.20		
	uniprobe mouse	UP00000_2	Smad3_secondary	3.64e-24	4.46e-21	8.13e-23	1639 / 2695 (60.8%)	1270 / 2695 (47.1%)	1.29	7.34		
	JASPAR2022 CORE vertebrates non-redundant	MA1596.1	MA1596.1.ZNF460	7.65e-24	9.37e-21	1.66e-22	467 / 2695 (17.3%)	223 / 2695 (8.3%)	2.09	5.03		
	uniprobe mouse	UP00068_2	Eomes_secondary	3.69e-23	4.51e-20	7.77e-22	2092 / 2695 (77.6%)	1766 / 2695 (65.5%)	1.18	5.29		
	JASPAR2022 CORE vertebrates non-redundant	MA2026.1	MA2026.1.CTCF	5.06e-23	6.20e-20	1.04e-21	1250 / 2695 (46.4%)	897 / 2695 (33.3%)	1.39	0.06		
	JASPAR2022 CORE vertebrates non-redundant	MA0073.1	MA0073.1.RREB1	5.43e-23	6.64e-20	1.08e-21	664 / 2695 (24.6%)	380 / 2695 (14.1%)	1.75	0.33		
	JASPAR2022 CORE vertebrates non-redundant	MA2032.1	MA2032.1.SP5	4.17e-22	5.10e-19	8.11e-21	819 / 2695 (30.4%)	515 / 2695 (19.1%)	1.59	7.25		
	JASPAR2022 CORE vertebrates non-redundant	MA1513.1	MA1513.1.KLF15	6.79e-22	8.31e-19	1.29e-20	966 / 2695 (35.8%)	645 / 2695 (23.9%)	1.50	3.33		
	JASPAR2022 CORE vertebrates non-redundant	MA2039.1	MA2039.1.Prdm5	7.78e-22	9.53e-19	1.44e-20	1335 / 2695 (49.5%)	988 / 2695 (36.7%)	1.35	7.85		
	uniprobe mouse	UP00036_2	Myf6_secondary	2.04e-21	2.50e-18	3.69e-20	2200 / 2695 (81.6%)	1905 / 2695 (70.7%)	1.15	6.68		
	JASPAR2022 CORE vertebrates non-redundant	MA0747.1	MA0747.1.SP8	5.52e-21	6.75e-18	9.74e-20	863 / 2695 (32.0%)	561 / 2695 (20.8%)	1.54	8.53		
	uniprobe mouse	UP00100_1	Gata6_primary	9.37e-21	1.15e-17	1.62e-19	754 / 2695 (28.0%)	469 / 2695 (17.4%)	1.61	10.01		
	JASPAR2022 CORE vertebrates non-redundant	MA1631.1	MA1631.1.ASCL1	1.92e-20	2.35e-17	3.23e-19	1029 / 2695 (38.2%)	713 / 2695 (26.5%)	1.44	7.76		
	JASPAR2022 CORE vertebrates non-redundant	MA0679.2	MA0679.2.ONECUT1	3.44e-20	4.21e-17	5.67e-19	1428 / 2695 (53.0%)	1093 / 2695 (40.6%)	1.31	7.31		
	JASPAR2022 CORE vertebrates non-redundant	MA1512.1	MA1512.1.KLF11	7.54e-20	9.23e-17	1.22e-18	798 / 2695 (29.6%)	513 / 2695 (19.0%)	1.55	7.74		
	uniprobe mouse	UP00099_2	Ascl2_secondary	8.70e-20	1.06e-16	1.38e-18	1504 / 2695 (55.8%)	1172 / 2695 (43.5%)	1.28	7.80		
	JASPAR2022 CORE vertebrates non-redundant	MA1485.1	MA1485.1.FERD3L	2.55e-19	3.12e-16	3.95e-18	843 / 2695 (31.3%)	556 / 2695 (20.6%)	1.52	0.01		
	JASPAR2022 CORE vertebrates non-redundant	MA1133.1	MA1133.1.JUN::JUNB	1.66e-18	2.03e-15	2.52e-17	295 / 2695 (10.9%)	125 / 2695 (4.6%)	2.35	10.94		
	uniprobe mouse	UP00084_2	Gmeb1_secondary	2.10e-18	2.58e-15	3.13e-17	1500 / 2695 (55.7%)	1181 / 2695 (43.8%)	1.27	7.67		
	JASPAR2022 CORE vertebrates non-redundant	MA0756.2	MA0756.2.ONECUT2	3.69e-18	4.52e-15	5.39e-17	819 / 2695 (30.4%)	544 / 2695 (20.2%)	1.50	8.79		
	JASPAR2022 CORE vertebrates non-redundant	MA0500.2	MA0500.2.MYOG	4.21e-18	5.15e-15	6.02e-17	1024 / 2695 (38.0%)	728 / 2695 (27.0%)	1.41	6.32		
	JASPAR2022 CORE vertebrates non-redundant	MA0753.2	MA0753.2.ZNF740	1.12e-17	1.37e-14	1.58e-16	1653 / 2695 (61.3%)	1343 / 2695 (49.8%)	1.23	1.03		
	JASPAR2022 CORE vertebrates non-redundant	MA0755.1	MA0755.1.CUX2	1.99e-17	2.44e-14	2.75e-16	649 / 2695 (24.1%)	404 / 2695 (15.0%)	1.60	10.03		
	JASPAR2022 CORE vertebrates non-redundant	MA0147.3	MA0147.3.MYC	2.06e-17	2.53e-14	2.80e-16	1120 / 2695 (41.6%)	823 / 2695 (30.5%)	1.36	7.00		
	JASPAR2022 CORE vertebrates non-redundant	MA1652.1	MA1652.1.ZKSCAN5	3.91e-17	4.79e-14	5.21e-16	1637 / 2695 (60.7%)	1332 / 2695 (49.4%)	1.23	5.65		
	JASPAR2022 CORE vertebrates non-redundant	MA1871.1	MA1871.1.ZFP14	3.99e-17	4.88e-14	5.22e-16	1702 / 2695 (63.2%)	1399 / 2695 (51.9%)	1.22	3.45		
	JASPAR2022 CORE vertebrates non-redundant	MA0732.1	MA0732.1.EGR3	4.26e-17	5.21e-14	5.47e-16	470 / 2695 (17.4%)	261 / 2695 (9.7%)	1.80	4.38		
	JASPAR2022 CORE vertebrates non-redundant	MA1475.1	MA1475.1.CREB3L4	1.88e-16	2.30e-13	2.38e-15	167 / 2695 (6.2%)	51 / 2695 (1.9%)	3.23	12.28		
	JASPAR2022 CORE vertebrates non-redundant	MA1140.2	MA1140.2.JUNB	2.78e-16	3.41e-13	3.46e-15	188 / 2695 (7.0%)	64 / 2695 (2.4%)	2.91	8.93		
	JASPAR2022 CORE vertebrates non-redundant	MA0522.3	MA0522.3.TCF3	3.45e-16	4.22e-13	4.22e-15	2236 / 2695 (83.0%)	1992 / 2695 (73.9%)	1.12	5.01		
	uniprobe mouse	UP00103_1	Jundm2_primary	3.69e-16	4.52e-13	4.45e-15	406 / 2695 (15.1%)	217 / 2695 (8.1%)	1.87	8.28		
	JASPAR2022 CORE vertebrates non-redundant	MA1710.1	MA1710.1.ZNF257	7.87e-16	9.64e-13	9.34e-15	622 / 2695 (23.1%)	393 / 2695 (14.6%)	1.58	8.26		
	JASPAR2022 CORE vertebrates non-redundant	MA1522.1	MA1522.1.MAZ	1.25e-15	1.53e-12	1.46e-14	728 / 2695 (27.0%)	485 / 2695 (18.0%)	1.50	6.24		
	JASPAR2022 CORE vertebrates non-redundant	MA0754.2	MA0754.2.CUX1	1.49e-15	1.82e-12	1.71e-14	952 / 2695 (35.3%)	685 / 2695 (25.4%)	1.39	8.70		
	JASPAR2022 CORE vertebrates non-redundant	MA2046.1	MA2046.1.Ikzf3	2.36e-15	2.89e-12	2.68e-14	1693 / 2695 (62.8%)	1408 / 2695 (52.2%)	1.20	6.12		
	JASPAR2022 CORE vertebrates non-redundant	MA0640.2	MA0640.2.ELF3	3.68e-15	4.50e-12	4.10e-14	1186 / 2695 (44.0%)	907 / 2695 (33.7%)	1.31	7.91		
	JASPAR2022 CORE vertebrates non-redundant	MA1515.1	MA1515.1.KLF2	5.42e-15	6.64e-12	5.96e-14	1353 / 2695 (50.2%)	1070 / 2695 (39.7%)	1.26	7.49		
	JASPAR2022 CORE vertebrates non-redundant	MA1136.1	MA1136.1.FOSB::JUNB	7.68e-15	9.40e-12	8.33e-14	172 / 2695 (6.4%)	59 / 2695 (2.2%)	2.88	12.18		
	JASPAR2022 CORE vertebrates non-redundant	MA0844.1	MA0844.1.XBP1	1.10e-14	1.35e-11	1.18e-13	966 / 2695 (35.8%)	706 / 2695 (26.2%)	1.37	5.17		
	JASPAR2022 CORE vertebrates non-redundant	MA1648.1	MA1648.1.TCF12	3.81e-14	4.67e-11	4.02e-13	1431 / 2695 (53.1%)	1156 / 2695 (42.9%)	1.24	7.63		
	JASPAR2022 CORE vertebrates non-redundant	MA1800.1	MA1800.1.FOS	4.39e-14	5.37e-11	4.56e-13	300 / 2695 (11.1%)	149 / 2695 (5.5%)	2.01	7.87		
	JASPAR2022 CORE vertebrates non-redundant	MA1799.1	MA1799.1.ETV5::FIGLA	4.74e-14	5.80e-11	4.86e-13	1437 / 2695 (53.3%)	1163 / 2695 (43.2%)	1.24	1.52		
	JASPAR2022 CORE vertebrates non-redundant	MA1719.1	MA1719.1.ZNF816	5.67e-14	6.93e-11	5.73e-13	2136 / 2695 (79.3%)	1899 / 2695 (70.5%)	1.12	0.47		
	JASPAR2022 CORE vertebrates non-redundant	MA1145.1	MA1145.1.FOSL2::JUND	1.07e-13	1.31e-10	1.07e-12	669 / 2695 (24.8%)	450 / 2695 (16.7%)	1.49	7.86		
	JASPAR2022 CORE vertebrates non-redundant	MA0136.3	MA0136.3.Elf5	1.09e-13	1.33e-10	1.07e-12	1277 / 2695 (47.4%)	1010 / 2695 (37.5%)	1.26	7.77		
	JASPAR2022 CORE vertebrates non-redundant	MA0609.2	MA0609.2.CREM	1.23e-13	1.51e-10	1.20e-12	189 / 2695 (7.0%)	74 / 2695 (2.7%)	2.53	11.33		
	JASPAR2022 CORE vertebrates non-redundant	MA1100.2	MA1100.2.ASCL1	1.53e-13	1.87e-10	1.47e-12	356 / 2695 (13.2%)	194 / 2695 (7.2%)	1.83	10.62		
	uniprobe mouse	UP00022_1	Zfp740_primary	1.67e-13	2.04e-10	1.58e-12	1500 / 2695 (55.7%)	1232 / 2695 (45.7%)	1.22	4.70		
	JASPAR2022 CORE vertebrates non-redundant	MA0638.1	MA0638.1.CREB3	1.88e-13	2.30e-10	1.76e-12	907 / 2695 (33.7%)	664 / 2695 (24.6%)	1.37	5.23		
	JASPAR2022 CORE vertebrates non-redundant	MA0780.1	MA0780.1.PAX3	1.99e-13	2.43e-10	1.84e-12	1528 / 2695 (56.7%)	1261 / 2695 (46.8%)	1.21	3.99		
	JASPAR2022 CORE vertebrates non-redundant	MA1131.1	MA1131.1.FOSL2::JUN	2.63e-13	3.22e-10	2.41e-12	84 / 2695 (3.1%)	15 / 2695 (0.6%)	5.31	14.39		
	JASPAR2022 CORE vertebrates non-redundant	MA1527.1	MA1527.1.NFIC	3.90e-13	4.78e-10	3.52e-12	41 / 2695 (1.5%)	0 / 2695 (0.0%)	42.00	19.24		
	JASPAR2022 CORE vertebrates non-redundant	MA0528.2	MA0528.2.ZNF263	7.49e-13	9.17e-10	6.69e-12	1954 / 2695 (72.5%)	1711 / 2695 (63.5%)	1.14	5.17		
	JASPAR2022 CORE vertebrates non-redundant	MA1127.1	MA1127.1.FOSB::JUN	8.91e-13	1.09e-9	7.86e-12	102 / 2695 (3.8%)	25 / 2695 (0.9%)	3.96	13.69		
	JASPAR2022 CORE vertebrates non-redundant	MA1528.1	MA1528.1.NFIX	1.10e-12	1.35e-9	9.63e-12	44 / 2695 (1.6%)	1 / 2695 (0.0%)	22.50	17.54		
	uniprobe mouse	UP00050_2	Bhlhb2_secondary	1.18e-12	1.44e-9	1.02e-11	851 / 2695 (31.6%)	621 / 2695 (23.0%)	1.37	5.83		
	JASPAR2022 CORE vertebrates non-redundant	MA0037.4	MA0037.4.Gata3	2.04e-12	2.50e-9	1.74e-11	455 / 2695 (16.9%)	280 / 2695 (10.4%)	1.62	10.55		
	uniprobe mouse	UP00046_2	Tcfe2a_secondary	2.19e-12	2.68e-9	1.83e-11	1479 / 2695 (54.9%)	1224 / 2695 (45.4%)	1.21	6.61		
	JASPAR2022 CORE vertebrates non-redundant	MA1102.2	MA1102.2.CTCFL	2.20e-12	2.69e-9	1.83e-11	1936 / 2695 (71.8%)	1697 / 2695 (63.0%)	1.14	3.87		
	uniprobe mouse	UP00020_1	Atf1_primary	3.43e-12	4.19e-9	2.83e-11	154 / 2695 (5.7%)	57 / 2695 (2.1%)	2.67	11.71		
	JASPAR2022 CORE vertebrates non-redundant	MA0471.2	MA0471.2.E2F6	4.91e-12	6.01e-9	4.01e-11	1312 / 2695 (48.7%)	1063 / 2695 (39.4%)	1.23	7.18		
	JASPAR2022 CORE vertebrates non-redundant	MA1730.1	MA1730.1.ZNF708	5.94e-12	7.27e-9	4.79e-11	1891 / 2695 (70.2%)	1654 / 2695 (61.4%)	1.14	2.02		
	uniprobe mouse	UP00219_2	Cutl1_3494.2	6.76e-12	8.27e-9	5.40e-11	1940 / 2695 (72.0%)	1707 / 2695 (63.3%)	1.14	6.42		
	JASPAR2022 CORE vertebrates non-redundant	MA0606.2	MA0606.2.Nfat5	6.87e-12	8.41e-9	5.43e-11	1477 / 2695 (54.8%)	1228 / 2695 (45.6%)	1.20	8.13		
	JASPAR2022 CORE vertebrates non-redundant	MA2021.1	MA2021.1.TRPS1	7.16e-12	8.77e-9	5.60e-11	382 / 2695 (14.2%)	225 / 2695 (8.3%)	1.69	11.05		
	JASPAR2022 CORE vertebrates non-redundant	MA1104.2	MA1104.2.GATA6	7.51e-12	9.19e-9	5.82e-11	413 / 2695 (15.3%)	250 / 2695 (9.3%)	1.65	11.02		
	JASPAR2022 CORE vertebrates non-redundant	MA0846.1	MA0846.1.FOXC2	8.55e-12	1.05e-8	6.56e-11	2057 / 2695 (76.3%)	1835 / 2695 (68.1%)	1.12	6.75		
	uniprobe mouse	UP00033_2	Zfp410_secondary	9.79e-12	1.20e-8	7.43e-11	1741 / 2695 (64.6%)	1499 / 2695 (55.6%)	1.16	7.37		
	JASPAR2022 CORE vertebrates non-redundant	MA0834.1	MA0834.1.ATF7	1.08e-11	1.32e-8	8.10e-11	95 / 2695 (3.5%)	24 / 2695 (0.9%)	3.84	11.10		
	JASPAR2022 CORE vertebrates non-redundant	MA1474.1	MA1474.1.CREB3L4	1.30e-11	1.59e-8	9.69e-11	794 / 2695 (29.5%)	580 / 2695 (21.5%)	1.37	8.95		
	JASPAR2022 CORE vertebrates non-redundant	MA0119.1	MA0119.1.NFIC::TLX1	1.66e-11	2.04e-8	1.22e-10	40 / 2695 (1.5%)	1 / 2695 (0.0%)	20.50	18.24		
	JASPAR2022 CORE vertebrates non-redundant	MA0816.1	MA0816.1.Ascl2	2.10e-11	2.57e-8	1.53e-10	313 / 2695 (11.6%)	174 / 2695 (6.5%)	1.79	10.31		
	JASPAR2022 CORE vertebrates non-redundant	MA1557.1	MA1557.1.SMAD5	2.18e-11	2.66e-8	1.57e-10	345 / 2695 (12.8%)	199 / 2695 (7.4%)	1.73	9.33		
	JASPAR2022 CORE vertebrates non-redundant	MA0604.1	MA0604.1.Atf1	3.44e-11	4.21e-8	2.46e-10	328 / 2695 (12.2%)	187 / 2695 (6.9%)	1.75	11.72		
	JASPAR2022 CORE vertebrates non-redundant	MA0733.1	MA0733.1.EGR4	5.48e-11	6.71e-8	3.89e-10	933 / 2695 (34.6%)	714 / 2695 (26.5%)	1.31	6.78		
	JASPAR2022 CORE vertebrates non-redundant	MA0656.1	MA0656.1.JDP2	5.71e-11	6.99e-8	4.01e-10	76 / 2695 (2.8%)	16 / 2695 (0.6%)	4.53	12.92		
	JASPAR2022 CORE vertebrates non-redundant	MA0033.2	MA0033.2.FOXL1	7.08e-11	8.66e-8	4.93e-10	1963 / 2695 (72.8%)	1744 / 2695 (64.7%)	1.13	7.15		
	JASPAR2022 CORE vertebrates non-redundant	MA0473.3	MA0473.3.ELF1	7.46e-11	9.13e-8	5.15e-10	1451 / 2695 (53.8%)	1215 / 2695 (45.1%)	1.19	6.43		
	JASPAR2022 CORE vertebrates non-redundant	MA0633.2	MA0633.2.Twist2	9.08e-11	1.11e-7	6.21e-10	741 / 2695 (27.5%)	541 / 2695 (20.1%)	1.37	5.38		
	JASPAR2022 CORE vertebrates non-redundant	MA1779.1	MA1779.1.TFAP4::ETV1	9.17e-11	1.12e-7	6.21e-10	2418 / 2695 (89.7%)	2259 / 2695 (83.8%)	1.07	1.13		
	JASPAR2022 CORE vertebrates non-redundant	MA0598.3	MA0598.3.EHF	9.37e-11	1.15e-7	6.29e-10	1118 / 2695 (41.5%)	891 / 2695 (33.1%)	1.25	7.87		
	JASPAR2022 CORE vertebrates non-redundant	MA1129.1	MA1129.1.FOSL1::JUN	9.60e-11	1.17e-7	6.39e-10	79 / 2695 (2.9%)	18 / 2695 (0.7%)	4.21	13.24		
	JASPAR2022 CORE vertebrates non-redundant	MA1517.1	MA1517.1.KLF6	1.11e-10	1.35e-7	7.30e-10	1971 / 2695 (73.1%)	1755 / 2695 (65.1%)	1.12	4.25		
	JASPAR2022 CORE vertebrates non-redundant	MA1651.1	MA1651.1.ZFP42	1.21e-10	1.48e-7	7.91e-10	807 / 2695 (29.9%)	602 / 2695 (22.3%)	1.34	6.98		
	JASPAR2022 CORE vertebrates non-redundant	MA0605.2	MA0605.2.ATF3	1.27e-10	1.56e-7	8.27e-10	88 / 2695 (3.3%)	23 / 2695 (0.9%)	3.71	10.60		

uniprobe mouse	UP00043_2	Bcl6b_secondary	1.42e-10	1.74e-7	9.15e-10	1233 / 2695 (45.8%)	1004 / 2695 (37.3%)	1.23	8.37
JASPAR2022 CORE vertebrates non-redundant	MA0840.1	MA0840.1.Creb5	1.57e-10	1.92e-7	1.00e-9	72 / 2695 (2.7%)	15 / 2695 (0.6%)	4.56	14.44
JASPAR2022 CORE vertebrates non-redundant	MA0139.1	MA0139.1.CTCF	2.78e-10	3.40e-7	1.76e-9	867 / 2695 (32.2%)	661 / 2695 (24.5%)	1.31	4.10
JASPAR2022 CORE vertebrates non-redundant	MA0845.1	MA0845.1.FOXB1	3.16e-10	3.87e-7	1.98e-9	2226 / 2695 (82.6%)	2041 / 2695 (75.7%)	1.09	5.57
JASPAR2022 CORE vertebrates non-redundant	MA1711.1	MA1711.1.ZNF343	3.72e-10	4.55e-7	2.31e-9	1025 / 2695 (38.0%)	810 / 2695 (30.1%)	1.27	0.04
JASPAR2022 CORE vertebrates non-redundant	MA1143.1	MA1143.1.FOSL1::JUND	5.07e-10	6.21e-7	3.13e-9	135 / 2695 (5.0%)	53 / 2695 (2.0%)	2.52	11.91
uniprobe mouse	UP00001_2	E2F2_secondary	6.36e-10	7.79e-7	3.89e-9	2222 / 2695 (82.4%)	2040 / 2695 (75.7%)	1.09	4.16
JASPAR2022 CORE vertebrates non-redundant	MA0839.1	MA0839.1.CREB3L1	6.71e-10	8.21e-7	4.07e-9	847 / 2695 (31.4%)	647 / 2695 (24.0%)	1.31	3.67
JASPAR2022 CORE vertebrates non-redundant	MA0830.2	MA0830.2.TCF4	7.05e-10	8.63e-7	4.25e-9	2536 / 2695 (94.1%)	2414 / 2695 (89.6%)	1.05	2.65
uniprobe mouse	UP00025_2	Foxk1_secondary	7.44e-10	9.11e-7	4.43e-9	1287 / 2695 (47.8%)	1066 / 2695 (39.6%)	1.21	8.08
uniprobe mouse	UP00003_2	E2F3_secondary	7.48e-10	9.16e-7	4.43e-9	969 / 2695 (36.0%)	761 / 2695 (28.2%)	1.27	7.55
JASPAR2022 CORE vertebrates non-redundant	MA1712.1	MA1712.1.ZNF454	7.92e-10	9.69e-7	4.66e-9	1929 / 2695 (71.6%)	1721 / 2695 (63.9%)	1.12	1.06
uniprobe mouse	UP00244_1	Tlx2_3498.2	9.33e-10	1.14e-6	5.45e-9	1371 / 2695 (50.9%)	1150 / 2695 (42.7%)	1.19	7.62
uniprobe mouse	UP00052_1	Osr2_primary	1.02e-9	1.25e-6	5.93e-9	485 / 2695 (18.0%)	327 / 2695 (12.1%)	1.48	8.57
JASPAR2022 CORE vertebrates non-redundant	MA1139.1	MA1139.1.FOSL2::JUNB	1.05e-9	1.28e-6	6.02e-9	78 / 2695 (2.9%)	20 / 2695 (0.7%)	3.76	13.90
JASPAR2022 CORE vertebrates non-redundant	MA1109.1	MA1109.1.NEUROD1	1.13e-9	1.38e-6	6.42e-9	287 / 2695 (10.6%)	165 / 2695 (6.1%)	1.73	11.03
JASPAR2022 CORE vertebrates non-redundant	MA1126.1	MA1126.1.FOS::JUN	1.37e-9	1.67e-6	7.75e-9	140 / 2695 (5.2%)	58 / 2695 (2.2%)	2.39	12.43
JASPAR2022 CORE vertebrates non-redundant	MA0741.1	MA0741.1.KLF16	1.42e-9	1.74e-6	7.98e-9	1813 / 2695 (67.3%)	1602 / 2695 (59.4%)	1.13	3.07
JASPAR2022 CORE vertebrates non-redundant	MA0486.2	MA0486.2.HSF1	1.96e-9	2.39e-6	1.09e-8	84 / 2695 (3.1%)	24 / 2695 (0.9%)	3.40	13.35
JASPAR2022 CORE vertebrates non-redundant	MA0668.2	MA0668.2.Neurod2	2.23e-9	2.73e-6	1.24e-8	711 / 2695 (26.4%)	529 / 2695 (19.6%)	1.34	8.62
uniprobe mouse	UP00036_1	Myf6_primary	2.43e-9	2.97e-6	1.33e-8	435 / 2695 (16.1%)	288 / 2695 (10.7%)	1.51	10.47
JASPAR2022 CORE vertebrates non-redundant	MA1684.1	MA1684.1.Foxn1	2.55e-9	3.12e-6	1.39e-8	1824 / 2695 (67.7%)	1617 / 2695 (60.0%)	1.13	8.52
JASPAR2022 CORE vertebrates non-redundant	MA0506.2	MA0506.2.Nrf1	3.19e-9	3.91e-6	1.73e-8	822 / 2695 (30.5%)	632 / 2695 (23.5%)	1.30	6.05
JASPAR2022 CORE vertebrates non-redundant	MA1650.1	MA1650.1.ZBTB14	3.98e-9	4.88e-6	2.14e-8	2174 / 2695 (80.7%)	1996 / 2695 (74.1%)	1.09	2.97
JASPAR2022 CORE vertebrates non-redundant	MA1646.1	MA1646.1.OSR2	4.03e-9	4.93e-6	2.15e-8	753 / 2695 (27.9%)	570 / 2695 (21.2%)	1.32	9.33
JASPAR2022 CORE vertebrates non-redundant	MA0482.2	MA0482.2.GATA4	4.09e-9	5.01e-6	2.17e-8	1546 / 2695 (57.4%)	1334 / 2695 (49.5%)	1.16	7.27
JASPAR2022 CORE vertebrates non-redundant	MA0499.2	MA0499.2.MYOD1	4.18e-9	5.11e-6	2.20e-8	959 / 2695 (35.6%)	761 / 2695 (28.2%)	1.26	7.76
JASPAR2022 CORE vertebrates non-redundant	MA1635.1	MA1635.1.BHLHE22	4.25e-9	5.21e-6	2.23e-8	449 / 2695 (16.7%)	302 / 2695 (11.2%)	1.49	6.22
JASPAR2022 CORE vertebrates non-redundant	MA0821.2	MA0821.2.HES5	4.88e-9	5.97e-6	2.54e-8	825 / 2695 (30.6%)	637 / 2695 (23.6%)	1.29	7.46
uniprobe mouse	UP00027_1	Osr1_primary	4.94e-9	6.05e-6	2.55e-8	1012 / 2695 (37.6%)	812 / 2695 (30.1%)	1.25	6.51
JASPAR2022 CORE vertebrates non-redundant	MA0048.2	MA0048.2.NHLH1	5.37e-9	6.57e-6	2.75e-8	386 / 2695 (14.3%)	250 / 2695 (9.3%)	1.54	8.80
uniprobe mouse	UP00013_2	Gabpa_secondary	1.20e-8	1.46e-5	6.10e-8	208 / 2695 (7.7%)	111 / 2695 (4.1%)	1.87	10.60
JASPAR2022 CORE vertebrates non-redundant	MA0032.2	MA0032.2.FOXC1	1.51e-8	1.85e-5	7.65e-8	1519 / 2695 (56.4%)	1315 / 2695 (48.8%)	1.16	7.74
JASPAR2022 CORE vertebrates non-redundant	MA0036.3	MA0036.3.GATA2	1.80e-8	2.20e-5	9.03e-8	1503 / 2695 (55.8%)	1300 / 2695 (48.2%)	1.16	7.76
JASPAR2022 CORE vertebrates non-redundant	MA2045.1	MA2045.1.Olig2	1.93e-8	2.36e-5	9.65e-8	493 / 2695 (18.3%)	346 / 2695 (12.8%)	1.42	7.68
uniprobe mouse	UP00042_2	Gm397_secondary	1.95e-8	2.39e-5	9.67e-8	391 / 2695 (14.5%)	259 / 2695 (9.6%)	1.51	9.51
JASPAR2022 CORE vertebrates non-redundant	MA0103.3	MA0103.3.ZEB1	2.10e-8	2.57e-5	1.03e-7	1592 / 2695 (59.1%)	1391 / 2695 (51.6%)	1.14	5.80
JASPAR2022 CORE vertebrates non-redundant	MA1554.1	MA1554.1.RFX7	2.58e-8	3.16e-5	1.26e-7	2048 / 2695 (76.0%)	1869 / 2695 (69.4%)	1.10	6.78
JASPAR2022 CORE vertebrates non-redundant	MA1632.1	MA1632.1.ATF2	2.59e-8	3.18e-5	1.26e-7	987 / 2695 (36.6%)	798 / 2695 (29.6%)	1.24	6.36
JASPAR2022 CORE vertebrates non-redundant	MA0161.2	MA0161.2.NFIC	4.26e-8	5.22e-5	2.06e-7	445 / 2695 (16.5%)	308 / 2695 (11.4%)	1.44	10.04
JASPAR2022 CORE vertebrates non-redundant	MA0062.3	MA0062.3.GABPA	4.80e-8	5.87e-5	2.30e-7	792 / 2695 (29.4%)	619 / 2695 (23.0%)	1.28	8.70
JASPAR2022 CORE vertebrates non-redundant	MA0159.1	MA0159.1.RARA::RXRA	4.93e-8	6.03e-5	2.35e-7	1699 / 2695 (63.0%)	1506 / 2695 (55.9%)	1.13	2.51
JASPAR2022 CORE vertebrates non-redundant	MA0748.2	MA0748.2.YY2	7.00e-8	8.56e-5	3.32e-7	1646 / 2695 (61.1%)	1454 / 2695 (54.0%)	1.13	6.45
JASPAR2022 CORE vertebrates non-redundant	MA0018.4	MA0018.4.CREB1	7.23e-8	8.85e-5	3.41e-7	257 / 2695 (9.5%)	154 / 2695 (5.7%)	1.66	11.22
JASPAR2022 CORE vertebrates non-redundant	MA0697.2	MA0697.2.Zic3	7.36e-8	9.01e-5	3.45e-7	625 / 2695 (23.2%)	469 / 2695 (17.4%)	1.33	9.13
JASPAR2022 CORE vertebrates non-redundant	MA2044.1	MA2044.1.Neurod2	7.56e-8	9.26e-5	3.52e-7	922 / 2695 (34.2%)	743 / 2695 (27.6%)	1.24	3.46
JASPAR2022 CORE vertebrates non-redundant	MA0820.1	MA0820.1.FIGLA	7.60e-8	9.30e-5	3.52e-7	701 / 2695 (26.0%)	538 / 2695 (20.0%)	1.30	9.13
JASPAR2022 CORE vertebrates non-redundant	MA0694.1	MA0694.1.ZBTB7B	7.84e-8	9.59e-5	3.60e-7	1124 / 2695 (41.7%)	936 / 2695 (34.7%)	1.20	3.62
JASPAR2022 CORE vertebrates non-redundant	MA0472.2	MA0472.2.EGR2	8.50e-8	1.04e-4	3.86e-7	548 / 2695 (20.3%)	401 / 2695 (14.9%)	1.37	7.05
uniprobe mouse	UP00025_1	Foxk1_primary	8.50e-8	1.04e-4	3.86e-7	2331 / 2695 (86.5%)	2189 / 2695 (81.2%)	1.06	3.40
JASPAR2022 CORE vertebrates non-redundant	MA1148.1	MA1148.1.PPARA::RXRA	1.20e-7	1.47e-4	5.43e-7	937 / 2695 (34.8%)	760 / 2695 (28.2%)	1.23	7.95
JASPAR2022 CORE vertebrates non-redundant	MA0649.1	MA0649.1.HEY2	1.38e-7	1.68e-4	6.18e-7	525 / 2695 (19.5%)	383 / 2695 (14.2%)	1.37	8.79
JASPAR2022 CORE vertebrates non-redundant	MA0745.2	MA0745.2.SNAI2	1.51e-7	1.84e-4	6.72e-7	926 / 2695 (34.4%)	751 / 2695 (27.9%)	1.23	6.68
JASPAR2022 CORE vertebrates non-redundant	MA1797.1	MA1797.1.ERF::NHLH1	1.56e-7	1.91e-4	6.91e-7	604 / 2695 (22.4%)	454 / 2695 (16.8%)	1.33	2.99
uniprobe mouse	UP00082_2	Zfp187_secondary	2.21e-7	2.70e-4	9.74e-7	1878 / 2695 (69.7%)	1702 / 2695 (63.2%)	1.10	5.52
JASPAR2022 CORE vertebrates non-redundant	MA0488.1	MA0488.1.JUN	2.41e-7	2.95e-4	1.06e-6	359 / 2695 (13.3%)	242 / 2695 (9.0%)	1.48	8.71
JASPAR2022 CORE vertebrates non-redundant	MA0014.3	MA0014.3.PAX5	2.67e-7	3.26e-4	1.16e-6	536 / 2695 (19.9%)	396 / 2695 (14.7%)	1.35	9.52
JASPAR2022 CORE vertebrates non-redundant	MA1516.1	MA1516.1.KLF3	2.77e-7	3.39e-4	1.20e-6	1960 / 2695 (72.7%)	1790 / 2695 (66.4%)	1.09	0.59
JASPAR2022 CORE vertebrates non-redundant	MA0521.2	MA0521.2.Tcf12	3.17e-7	3.88e-4	1.37e-6	1994 / 2695 (74.0%)	1827 / 2695 (67.8%)	1.09	3.61
JASPAR2022 CORE vertebrates non-redundant	MA1587.1	MA1587.1.ZNF135	3.27e-7	4.00e-4	1.40e-6	924 / 2695 (34.3%)	754 / 2695 (28.0%)	1.23	3.56
JASPAR2022 CORE vertebrates non-redundant	MA0504.1	MA0504.1.NR2C2	3.29e-7	4.02e-4	1.40e-6	1140 / 2695 (42.3%)	961 / 2695 (35.7%)	1.19	3.89
uniprobe mouse	UP00071_1	Sox21_primary	4.15e-7	5.08e-4	1.76e-6	1930 / 2695 (71.6%)	1761 / 2695 (65.3%)	1.10	6.26
JASPAR2022 CORE vertebrates non-redundant	MA0680.2	MA0680.2.Pax7	4.35e-7	5.33e-4	1.83e-6	1976 / 2695 (73.3%)	1810 / 2695 (67.2%)	1.09	5.73
uniprobe mouse	UP00032_1	Gata3_primary	4.48e-7	5.49e-4	1.88e-6	1759 / 2695 (65.3%)	1583 / 2695 (58.7%)	1.11	5.16
JASPAR2022 CORE vertebrates non-redundant	MA1542.1	MA1542.1.OSR1	5.44e-7	6.66e-4	2.27e-6	1092 / 2695 (40.5%)	918 / 2695 (34.1%)	1.19	6.60
uniprobe mouse	UP00096_2	Sox13_secondary	5.73e-7	7.01e-4	2.38e-6	1211 / 2695 (44.9%)	1034 / 2695 (38.4%)	1.17	8.18
JASPAR2022 CORE vertebrates non-redundant	MA0664.1	MA0664.1.MLXIPL	6.51e-7	7.97e-4	2.68e-6	580 / 2695 (21.5%)	440 / 2695 (16.3%)	1.32	7.96
JASPAR2022 CORE vertebrates non-redundant	MA0851.1	MA0851.1.Foxj3	7.27e-7	8.90e-4	2.98e-6	2258 / 2695 (83.8%)	2119 / 2695 (78.6%)	1.07	4.12
uniprobe mouse	UP00049_2	Sp100_secondary	7.53e-7	9.22e-4	3.07e-6	1333 / 2695 (49.5%)	1156 / 2695 (42.9%)	1.15	8.06
JASPAR2022 CORE vertebrates non-redundant	MA1715.1	MA1715.1.ZNF707	8.59e-7	1.05e-3	3.48e-6	857 / 2695 (31.8%)	697 / 2695 (25.9%)	1.23	2.70
JASPAR2022 CORE vertebrates non-redundant	MA0771.1	MA0771.1.HSF4	9.02e-7	1.10e-3	3.64e-6	70 / 2695 (2.6%)	24 / 2695 (0.9%)	2.84	14.46
uniprobe mouse	UP00065_1	Zfp161_primary	9.38e-7	1.15e-3	3.77e-6	174 / 2695 (6.5%)	97 / 2695 (3.6%)	1.79	11.78
JASPAR2022 CORE vertebrates non-redundant	MA1599.1	MA1599.1.ZNF682	9.80e-7	1.20e-3	3.92e-6	111 / 2695 (4.1%)	51 / 2695 (1.9%)	2.15	11.92
JASPAR2022 CORE vertebrates non-redundant	MA1558.1	MA1558.1.SNAI1	1.00e-6	1.23e-3	3.98e-6	2122 / 2695 (78.7%)	1972 / 2695 (73.2%)	1.08	4.64
JASPAR2022 CORE vertebrates non-redundant	MA0104.4	MA0104.4.MYCN	1.24e-6	1.51e-3	4.89e-6	1005 / 2695 (37.3%)	840 / 2695 (31.2%)	1.20	5.84
JASPAR2022 CORE vertebrates non-redundant	MA0901.2	MA0901.2.HOXB13	1.49e-6	1.82e-3	5.85e-6	311 / 2695 (11.5%)	209 / 2695 (7.8%)	1.49	11.02
JASPAR2022 CORE vertebrates non-redundant	MA1642.1	MA1642.1.NEUROG2	1.60e-6	1.96e-3	6.26e-6	708 / 2695 (26.3%)	562 / 2695 (20.9%)	1.26	8.91
JASPAR2022 CORE vertebrates non-redundant	MA1532.2	MA1532.2.NR1D2	1.69e-6	2.07e-3	6.58e-6	278 / 2695 (10.3%)	182 / 2695 (6.8%)	1.52	2.70
uniprobe mouse	UP00114_1	Homez_1063.2	1.74e-6	2.13e-3	6.75e-6	946 / 2695 (35.1%)	786 / 2695 (29.2%)	1.20	8.68
JASPAR2022 CORE vertebrates non-redundant	MA1641.1	MA1641.1.MYF5	1.82e-6	2.23e-3	7.03e-6	196 / 2695 (7.3%)	116 / 2695 (4.3%)	1.68	10.55
JASPAR2022 CORE vertebrates non-redundant	MA0474.3	MA0474.3.Erg	2.10e-6	2.57e-3	8.05e-6	1233 / 2695 (45.8%)	1065 / 2695 (39.5%)	1.16	7.27
uniprobe mouse	UP00039_1	Foxj3_primary	2.43e-6	2.97e-3	9.26e-6	1747 / 2695 (64.8%)	1583 / 2695 (58.7%)	1.10	6.12
uniprobe mouse	UP00060_1	Max_primary	2.53e-6	3.10e-3	9.61e-6	2190 / 2695 (81.3%)	2052 / 2695 (76.1%)	1.07	2.69
JASPAR2022 CORE vertebrates non-redundant	MA0770.1	MA0770.1.HSF2	3.23e-6	3.95e-3	1.22e-5	241 / 2695 (8.9%)	154 / 2695 (5.7%)	1.56	10.37
uniprobe mouse	UP00095_1	Zfp691_primary	3.44e-6	4.21e-3	1.29e-5	1401 / 2695 (52.0%)	1235 / 2695 (45.8%)	1.13	3.84
uniprobe mouse	UP00255_1	Dbx1_3486.1	3.45e-6	4.22e-3	1.29e-5	584 / 2695 (21.7%)	453 / 2695 (16.8%)	1.29	8.87
uniprobe mouse	UP00037_1	Zfp105_primary	3.83e-6	4.69e-3	1.42e-5	1892 / 2695 (70.2%)	1737 / 2695 (64.5%)	1.09	7.34
JASPAR2022 CORE vertebrates non-redundant	MA2033.1	MA2033.1.THRA	4.09e-6	5.00e-3	1.51e-5	847 / 2695 (31.4%)	698 / 2695 (25.9%)	1.21	1.85
JASPAR2022 CORE vertebrates non-redundant	MA0492.1	MA0492.1.JUND	4.12e-6	5.05e-3	1.52e-5	42 / 2695 (1.6%)	10 / 2695 (0.4%)	3.91	15.72
JASPAR2022 CORE vertebrates non-redundant	MA1470.1	MA1470.1.BACH2	4.42e-6	5.41e-3	1.62e-5	205 / 2695 (7.6%)	126 / 2695 (4.7%)	1.62	0.70
JASPAR2022 CORE vertebrates non-redundant	MA1868.1	MA1868.1.ZNF320	4.65e-6	5.69e-3	1.69e-5	102 / 2695 (3.8%)	48 / 2695 (1.8%)	2.10	11.08
uniprobe mouse	UP00088_1	Plagl1_primary	4.88e-6	5.97e-3	1.77e-5	1992 / 2695 (73.9%)	1844 / 2695 (68.4%)	1.08	1.97
JASPAR2022 CORE vertebrates non-redundant	MA0671.1	MA0671.1.NFIX	5.28e-6	6.46e-3	1.91e-5	203 / 2695 (7.5%)	125 / 2695 (4.6%)	1.62	11.25
JASPAR2022 CORE vertebrates non-redundant	MA0052.4	MA0052.4.MEF2A	5.42e-6	6.64e-3	1.95e-5	925 / 2695 (34.3%)	774 / 2695 (28.7%)	1.19	8.71
uniprobe mouse	UP00053_2	Rxra_secondary	5.53e-6	6.77e-3	1.98e-5	1938 / 2695 (71.9%)	1788 / 2695 (66.3%)	1.08	7.11
JASPAR2022 CORE vertebrates non-redundant	MA0059.1	MA0059.1.MAX::MYC	6.29e-6	7.70e-3	2.24e-5	468 / 2695 (17.4%)	352 / 2695 (13.1%)	1.33	7.94
JASPAR2022 CORE vertebrates non-redundant	MA1529.1	MA1529.1.NHLH2	6.62e-6	8.10e-3	2.35e-5	85 / 2695 (3.2%)	37 / 2695 (1.4%)	2.26	10.25
JASPAR2022 CORE vertebrates non-redundant	MA1110.2	MA1110.2.Nr1H4	6.76e-6	8.27e-3	2.39e-5	1302 / 2695 (48.3%)	1142 / 2695 (42.4%)	1.14	7.20
JASPAR2022 CORE vertebrates non-redundant	MA1472.2	MA1472.2.Bhlha15	7.17e-6	8.78e-3	2.52e-5	116 / 2695 (4.3%)	59 / 2695 (2.2%)	1.95	11.98
uniprobe mouse	UP00076_2	Rfxdc2_secondary	7.48e-6	9.15e-3	2.61e-5	1633 / 2695 (60.6%)	1475 / 2695 (54.7%)	1.11	6.66
uniprobe mouse	UP00076_1	Rfxdc2_primary	7.52e-6	9.20e-3	2.62e-5	1006 / 2695 (37.3%)	854 / 2695 (31.7%)	1.18	6.35
JASPAR2022 CORE vertebrates non-redundant	MA1623.1	MA1623.1.Stat2	7.70e-6	9.42e-3	2.67e-5	2196 / 2695 (81.5%)	2066 / 2695 (76.7%)	1.06	5.99
JASPAR2022 CORE vertebrates non-redundant	MA1600.1	MA1600.1.ZNF684	8.04e-6	9.84e-3	2.77e-5	645 / 2695 (23.9%)	514 / 2695 (19.1%)	1.25	2.64
JASPAR2022 CORE vertebrates non-redundant	MA0466.3	MA0466.3.CEBPB	8.71e-6	1.07e-2	2.99e-5	269 / 2695 (10.0%)	181 / 2695 (6.7%)	1.48	6.11
JASPAR2022 CORE vertebrates non-redundant	MA1149.1	MA1149.1.RARA::RXRG	8.83e-6	1.08e-2	3.02e-5	2334 / 2695 (86.6%)	2219 / 2695 (82.3%)	1.05	2.46
JASPAR2022 CORE vertebrates non-redundant	MA1550.1	MA1550.1.PPARD	8.98e-6	1.10e-2	3.06e-5	581 / 2695 (21.6%)	456 / 2695 (16.9%)	1.27	2.30
JASPAR2022 CORE vertebrates non-redundant	MA0909.3	MA0909.3.Hoxd13	9.46e-6	1.16e-2	3.21e-5	1638 / 2695 (60.8%)	1482 / 2695 (55.0%)	1.11	5.99
uniprobe mouse	UP00026_1	Zscan4_primary	9.96e-6	1.22e-2	3.36e-5	637 / 2695 (23.6%)	508 / 2695 (18.8%)	1.25	7.54
JASPAR2022 CORE vertebrates non-redundant	MA1731.1	MA1731.1.ZNF768	1.10e-5	1.35e-2	3.70e-5	2480 / 2695 (92.0%)	2387 / 2695 (88.6%)	1.04	0.50
uniprobe mouse	UP00002_2	Sp4_secondary	1.26e-5	1.54e-2	4.21e-5	1134 / 2695 (42.1%)	982 / 2695 (36.4%)	1.15	7.36
JASPAR2022 CORE vertebrates non-redundant	MA1514.1	MA1514.1.KLF17	1.62e-5	1.98e-2	5.39e-5	182 / 2695 (6.8%)	112 / 2695 (4.2%)	1.62	10.02
JASPAR2022 CORE vertebrates non-redundant	MA1574.1	MA1574.1.THRB	1.64e-5	2.01e-2	5.45e-5	533 / 2695 (19.8%)	416 / 2695 (15.4%)	1.28	6.91
JASPAR2022 CORE vertebrates non-redundant	MA0829.2	MA0829.2.SREBF1	1.68e-5	2.06e-2	5.55e-5	1946 / 2695 (72.2%)	1805 / 2695 (67.0%)	1.08	3.16
JASPAR2022 CORE vertebrates non-redundant	MA1122.1	MA1122.1.TFDP1	1.89e-5	2.32e-2	6.22e-5	1857 / 2695 (68.9%)	1713 / 2695 (63.6%)	1.08	5.60
JASPAR2022 CORE vertebrates non-redundant	MA0076.2	MA0076.2.ELK4	1.91e-5	2.34e-2	6.26e-5	1475 / 2695 (54.7%)	1323 / 2695 (49.1%)	1.11	4.00
JASPAR2022 CORE vertebrates non-redundant	MA1487.2	MA1487.2.FOXE1	1.99e-5	2.44e-2	6.50e-5	2473 / 2695 (91.8%)	2382 / 2695 (88.4%)	1.04	2.61
JASPAR2022 CORE vertebrates non-redundant	MA1638.1	MA1638.1.HAND2	2.10e-5	2.58e-2	6.82e-5	1034 / 2695 (38.4%)	889 / 2695 (33.0%)	1.16	7.52
JASPAR2022 CORE vertebrates non-redundant	MA1573.2	MA1573.2.Thap11	2.18e-5	2.66e-2	7.01e-5	1945 / 2695 (72.2%)	1806 / 2695 (67.0%)	1.08	1.16
JASPAR2022 CORE vertebrates non-redundant	MA0681.2	MA0681.2.PHOX2B	2.18e-5	2.67e-2	7.01e-5	214 / 2695 (7.9%)	139 / 2695 (5.2%)	1.54	11.56
uniprobe mouse	UP00046_1	Tcfe2a_primary	2.27e-5	2.78e-2	7.26e-5	2618 / 2695 (97.1%)	2559 / 2695 (95.0%)	1.02	0.11

JASPAR2022 CORE vertebrates non-redundant	MA1869.1	MA1869.1.ZNF324	2.31e-5	2.83e-2	7.37e-5	899 / 2695 (33.4%)	760 / 2695 (28.2%)	1.18	4.81
uniprobe mouse	UP00031_1	Zbtb3_primary	2.37e-5	2.90e-2	7.53e-5	803 / 2695 (29.8%)	669 / 2695 (24.8%)	1.20	7.66
JASPAR2022 CORE vertebrates non-redundant	MA0616.2	MA0616.2.HES2	2.41e-5	2.95e-2	7.62e-5	1831 / 2695 (67.9%)	1688 / 2695 (62.6%)	1.08	1.76
JASPAR2022 CORE vertebrates non-redundant	MA0696.1	MA0696.1.ZIC1	2.61e-5	3.20e-2	8.22e-5	590 / 2695 (21.9%)	471 / 2695 (17.5%)	1.25	1.14
uniprobe mouse	UP00047_2	Zbtb7b_secondary	2.72e-5	3.32e-2	8.52e-5	2565 / 2695 (95.2%)	2493 / 2695 (92.5%)	1.03	1.11
JASPAR2022 CORE vertebrates non-redundant	MA1628.1	MA1628.1.Zic1::Zic2	2.91e-5	3.56e-2	9.07e-5	1022 / 2695 (37.9%)	880 / 2695 (32.7%)	1.16	7.05
JASPAR2022 CORE vertebrates non-redundant	MA0766.2	MA0766.2.GATA5	3.00e-5	3.67e-2	9.33e-5	1656 / 2695 (61.4%)	1510 / 2695 (56.0%)	1.10	5.96
JASPAR2022 CORE vertebrates non-redundant	MA0657.1	MA0657.1.KLF13	3.37e-5	4.13e-2	1.04e-4	70 / 2695 (2.6%)	30 / 2695 (1.1%)	2.29	9.59
JASPAR2022 CORE vertebrates non-redundant	MA0761.2	MA0761.2.ETV1	3.41e-5	4.18e-2	1.05e-4	1650 / 2695 (61.2%)	1505 / 2695 (55.8%)	1.10	6.24
JASPAR2022 CORE vertebrates non-redundant	MA0135.1	MA0135.1.Lhx3	3.47e-5	4.25e-2	1.07e-4	1411 / 2695 (52.4%)	1264 / 2695 (46.9%)	1.12	4.80
JASPAR2022 CORE vertebrates non-redundant	MA0750.2	MA0750.2.ZBTB7A	3.55e-5	4.35e-2	1.09e-4	2316 / 2695 (85.9%)	2208 / 2695 (81.9%)	1.05	4.03
JASPAR2022 CORE vertebrates non-redundant	MA0145.2	MA0145.2.Tfcp2l1	3.68e-5	4.51e-2	1.12e-4	2384 / 2695 (88.5%)	2284 / 2695 (84.7%)	1.04	2.12
JASPAR2022 CORE vertebrates non-redundant	MA0526.4	MA0526.4.USF2	4.08e-5	4.99e-2	1.24e-4	629 / 2695 (23.3%)	510 / 2695 (18.9%)	1.23	0.22
uniprobe mouse	UP00065_2	Zfp161_secondary	4.14e-5	5.06e-2	1.25e-4	356 / 2695 (13.2%)	263 / 2695 (9.8%)	1.35	10.30
JASPAR2022 CORE vertebrates non-redundant	MA0823.1	MA0823.1.HEY1	4.20e-5	5.14e-2	1.26e-4	520 / 2695 (19.3%)	410 / 2695 (15.2%)	1.27	7.77
uniprobe mouse	UP00098_2	Rfx3_secondary	4.22e-5	5.17e-2	1.27e-4	310 / 2695 (11.5%)	223 / 2695 (8.3%)	1.39	10.19
uniprobe mouse	UP00020_2	Atf1_secondary	4.25e-5	5.20e-2	1.27e-4	1345 / 2695 (49.9%)	1200 / 2695 (44.5%)	1.12	8.10
JASPAR2022 CORE vertebrates non-redundant	MA1729.1	MA1729.1.ZNF680	4.35e-5	5.32e-2	1.29e-4	1693 / 2695 (62.8%)	1551 / 2695 (57.6%)	1.09	4.48
JASPAR2022 CORE vertebrates non-redundant	MA0799.2	MA0799.2.RFX4	4.85e-5	5.94e-2	1.44e-4	124 / 2695 (4.6%)	70 / 2695 (2.6%)	1.76	5.16
JASPAR2022 CORE vertebrates non-redundant	MA0130.1	MA0130.1.ZNF354C	5.22e-5	6.39e-2	1.54e-4	1844 / 2695 (68.4%)	1708 / 2695 (63.4%)	1.08	8.35
uniprobe mouse	UP00007_1	Egr1_primary	6.03e-5	7.38e-2	1.77e-4	2391 / 2695 (88.7%)	2295 / 2695 (85.2%)	1.04	0.01
uniprobe mouse	UP00081_2	Mybl1_secondary	6.04e-5	7.40e-2	1.77e-4	272 / 2695 (10.1%)	192 / 2695 (7.1%)	1.41	10.94
uniprobe mouse	UP00041_2	Foxj1_secondary	6.75e-5	8.26e-2	1.97e-4	963 / 2695 (35.7%)	830 / 2695 (30.8%)	1.16	8.77
JASPAR2022 CORE vertebrates non-redundant	MA0067.2	MA0067.2.PAX2	6.87e-5	8.41e-2	2.00e-4	1099 / 2695 (40.8%)	962 / 2695 (35.7%)	1.14	2.54
JASPAR2022 CORE vertebrates non-redundant	MA1559.1	MA1559.1.SNAI3	7.13e-5	8.72e-2	2.06e-4	2232 / 2695 (82.8%)	2121 / 2695 (78.7%)	1.05	0.13
uniprobe mouse	UP00060_2	Max_secondary	7.80e-5	9.55e-2	2.25e-4	1202 / 2695 (44.6%)	1064 / 2695 (39.5%)	1.13	7.85
JASPAR2022 CORE vertebrates non-redundant	MA0790.1	MA0790.1.POU4F1	8.71e-5	1.07e-1	2.50e-4	649 / 2695 (24.1%)	534 / 2695 (19.8%)	1.21	9.08
uniprobe mouse	UP00001_1	E2F2_primary	9.09e-5	1.11e-1	2.60e-4	669 / 2695 (24.8%)	553 / 2695 (20.5%)	1.21	8.47
JASPAR2022 CORE vertebrates non-redundant	MA0795.1	MA0795.1.SMAD3	9.13e-5	1.12e-1	2.60e-4	1120 / 2695 (41.6%)	985 / 2695 (36.5%)	1.14	1.98
JASPAR2022 CORE vertebrates non-redundant	MA0736.1	MA0736.1.GLIS2	9.56e-5	1.17e-1	2.72e-4	1095 / 2695 (40.6%)	961 / 2695 (35.7%)	1.14	0.47
JASPAR2022 CORE vertebrates non-redundant	MA1643.1	MA1643.1.NFIB	9.83e-5	1.20e-1	2.78e-4	64 / 2695 (2.4%)	28 / 2695 (1.0%)	2.24	12.39
JASPAR2022 CORE vertebrates non-redundant	MA0065.2	MA0065.2.Pparg::Rxra	1.01e-4	1.24e-1	2.85e-4	2212 / 2695 (82.1%)	2102 / 2695 (78.0%)	1.05	4.84
JASPAR2022 CORE vertebrates non-redundant	MA0670.1	MA0670.1.NFIA	1.05e-4	1.28e-1	2.95e-4	175 / 2695 (6.5%)	113 / 2695 (4.2%)	1.54	12.91
JASPAR2022 CORE vertebrates non-redundant	MA0815.1	MA0815.1.TFAP2C	1.10e-4	1.34e-1	3.07e-4	293 / 2695 (10.9%)	213 / 2695 (7.9%)	1.37	2.72
JASPAR2022 CORE vertebrates non-redundant	MA0111.1	MA0111.1.Spz1	1.14e-4	1.40e-1	3.19e-4	2381 / 2695 (88.3%)	2288 / 2695 (84.9%)	1.04	3.60
JASPAR2022 CORE vertebrates non-redundant	MA0715.1	MA0715.1.PROP1	1.29e-4	1.58e-1	3.58e-4	2130 / 2695 (79.0%)	2016 / 2695 (74.8%)	1.06	3.55
JASPAR2022 CORE vertebrates non-redundant	MA0837.2	MA0837.2.CEBPE	1.37e-4	1.67e-1	3.78e-4	175 / 2695 (6.5%)	114 / 2695 (4.2%)	1.53	8.38
JASPAR2022 CORE vertebrates non-redundant	MA1467.2	MA1467.2.Atoh1	1.40e-4	1.71e-1	3.86e-4	103 / 2695 (3.8%)	57 / 2695 (2.1%)	1.79	13.39
JASPAR2022 CORE vertebrates non-redundant	MA0734.3	MA0734.3.Gli2	1.47e-4	1.80e-1	4.05e-4	1691 / 2695 (62.7%)	1560 / 2695 (57.9%)	1.08	3.73
JASPAR2022 CORE vertebrates non-redundant	MA1620.1	MA1620.1.Ptf1A	1.51e-4	1.85e-1	4.14e-4	357 / 2695 (13.2%)	271 / 2695 (10.1%)	1.32	10.16
JASPAR2022 CORE vertebrates non-redundant	MA0819.2	MA0819.2.CLOCK	1.63e-4	2.00e-1	4.45e-4	981 / 2695 (36.4%)	855 / 2695 (31.7%)	1.15	7.23
JASPAR2022 CORE vertebrates non-redundant	MA0608.1	MA0608.1.Creb3l2	1.66e-4	2.03e-1	4.50e-4	2144 / 2695 (79.6%)	2033 / 2695 (75.4%)	1.05	0.28
JASPAR2022 CORE vertebrates non-redundant	MA1521.1	MA1521.1.MAFA	1.76e-4	2.15e-1	4.77e-4	2053 / 2695 (76.2%)	1937 / 2695 (71.9%)	1.06	3.87
JASPAR2022 CORE vertebrates non-redundant	MA0872.1	MA0872.1.TFAP2A	2.16e-4	2.64e-1	5.82e-4	322 / 2695 (11.9%)	242 / 2695 (9.0%)	1.33	3.98
JASPAR2022 CORE vertebrates non-redundant	MA1466.1	MA1466.1.ATF6	2.47e-4	3.03e-1	6.65e-4	587 / 2695 (21.8%)	484 / 2695 (18.0%)	1.21	0.08
JASPAR2022 CORE vertebrates non-redundant	MA1647.2	MA1647.2.Prdm4	2.65e-4	3.24e-1	7.07e-4	2217 / 2695 (82.3%)	2115 / 2695 (78.5%)	1.05	2.96
JASPAR2022 CORE vertebrates non-redundant	MA0626.1	MA0626.1.Npas2	2.65e-4	3.24e-1	7.07e-4	1595 / 2695 (59.2%)	1468 / 2695 (54.5%)	1.09	4.41
JASPAR2022 CORE vertebrates non-redundant	MA1153.1	MA1153.1.Smad4	2.99e-4	3.66e-1	7.96e-4	137 / 2695 (5.1%)	86 / 2695 (3.2%)	1.59	13.30
uniprobe mouse	UP00100_2	Gata6_secondary	3.02e-4	3.70e-1	8.02e-4	1953 / 2695 (72.5%)	1837 / 2695 (68.2%)	1.06	5.13
uniprobe mouse	UP00034_2	Sox7_secondary	3.25e-4	3.97e-1	8.58e-4	481 / 2695 (17.8%)	388 / 2695 (14.4%)	1.24	9.73
JASPAR2022 CORE vertebrates non-redundant	MA1708.1	MA1708.1.ETV7	3.30e-4	4.03e-1	8.68e-4	904 / 2695 (33.5%)	787 / 2695 (29.2%)	1.15	8.95
JASPAR2022 CORE vertebrates non-redundant	MA1807.1	MA1807.1.HOXD12::ELK1	3.34e-4	4.08e-1	8.76e-4	638 / 2695 (23.7%)	534 / 2695 (19.8%)	1.19	0.19
JASPAR2022 CORE vertebrates non-redundant	MA0838.1	MA0838.1.CEBPG	3.53e-4	4.33e-1	9.25e-4	93 / 2695 (3.5%)	52 / 2695 (1.9%)	1.77	11.55
JASPAR2022 CORE vertebrates non-redundant	MA0783.1	MA0783.1.PKNOX2	3.58e-4	4.38e-1	9.33e-4	330 / 2695 (12.2%)	252 / 2695 (9.4%)	1.31	0.24
JASPAR2022 CORE vertebrates non-redundant	MA1801.1	MA1801.1.FOXJ2::ELF1	3.79e-4	4.63e-1	9.84e-4	1684 / 2695 (62.5%)	1562 / 2695 (58.0%)	1.08	6.54
JASPAR2022 CORE vertebrates non-redundant	MA1629.1	MA1629.1.Zic2	3.90e-4	4.78e-1	1.01e-3	537 / 2695 (19.9%)	441 / 2695 (16.4%)	1.22	8.39
JASPAR2022 CORE vertebrates non-redundant	MA0773.1	MA0773.1.MEF2D	3.92e-4	4.80e-1	1.01e-3	921 / 2695 (34.2%)	805 / 2695 (29.9%)	1.14	0.75
JASPAR2022 CORE vertebrates non-redundant	MA0058.3	MA0058.3.MAX	3.94e-4	4.82e-1	1.01e-3	103 / 2695 (3.8%)	60 / 2695 (2.2%)	1.70	13.26
JASPAR2022 CORE vertebrates non-redundant	MA0877.3	MA0877.3.BARHL1	3.96e-4	4.85e-1	1.02e-3	2688 / 2695 (99.7%)	2668 / 2695 (99.0%)	1.01	2.52
JASPAR2022 CORE vertebrates non-redundant	MA0691.1	MA0691.1.TFAP4	4.02e-4	4.93e-1	1.03e-3	1238 / 2695 (45.9%)	1115 / 2695 (41.4%)	1.11	1.96
JASPAR2022 CORE vertebrates non-redundant	MA2038.1	MA2038.1.Gli1	4.14e-4	5.07e-1	1.05e-3	784 / 2695 (29.1%)	674 / 2695 (25.0%)	1.16	7.74
JASPAR2022 CORE vertebrates non-redundant	MA0164.1	MA0164.1.Nr2e3	4.72e-4	5.78e-1	1.20e-3	418 / 2695 (15.5%)	333 / 2695 (12.4%)	1.25	10.39
JASPAR2022 CORE vertebrates non-redundant	MA0108.2	MA0108.2.TBP	5.08e-4	6.21e-1	1.28e-3	780 / 2695 (28.9%)	672 / 2695 (24.9%)	1.16	9.02
JASPAR2022 CORE vertebrates non-redundant	MA0046.2	MA0046.2.HNF1A	5.25e-4	6.42e-1	1.32e-3	1922 / 2695 (71.3%)	1810 / 2695 (67.2%)	1.06	3.35
JASPAR2022 CORE vertebrates non-redundant	MA1717.1	MA1717.1.ZNF784	5.31e-4	6.49e-1	1.33e-3	324 / 2695 (12.0%)	249 / 2695 (9.2%)	1.30	9.68
JASPAR2022 CORE vertebrates non-redundant	MA1619.1	MA1619.1.Ptf1A	5.40e-4	6.60e-1	1.35e-3	1887 / 2695 (70.0%)	1774 / 2695 (65.8%)	1.06	3.63
JASPAR2022 CORE vertebrates non-redundant	MA0848.1	MA0848.1.FOXO4	5.66e-4	6.93e-1	1.41e-3	1529 / 2695 (56.7%)	1409 / 2695 (52.3%)	1.09	7.08
JASPAR2022 CORE vertebrates non-redundant	MA0100.3	MA0100.3.MYB	5.69e-4	6.96e-1	1.42e-3	852 / 2695 (31.6%)	742 / 2695 (27.5%)	1.15	8.67
JASPAR2022 CORE vertebrates non-redundant	MA0751.1	MA0751.1.ZIC4	6.31e-4	7.72e-1	1.56e-3	334 / 2695 (12.4%)	259 / 2695 (9.6%)	1.29	9.29
JASPAR2022 CORE vertebrates non-redundant	MA0095.3	MA0095.3.Yy1	6.48e-4	7.93e-1	1.60e-3	326 / 2695 (12.1%)	252 / 2695 (9.4%)	1.29	10.79
uniprobe mouse	UP00027_2	Osr1_secondary	6.61e-4	8.09e-1	1.63e-3	1644 / 2695 (61.0%)	1527 / 2695 (56.7%)	1.08	7.25
JASPAR2022 CORE vertebrates non-redundant	MA0041.2	MA0041.2.FOXD3	6.86e-4	8.40e-1	1.69e-3	774 / 2695 (28.7%)	669 / 2695 (24.8%)	1.16	9.22
JASPAR2022 CORE vertebrates non-redundant	MA0646.1	MA0646.1.GCM1	7.84e-4	9.60e-1	1.92e-3	1586 / 2695 (58.8%)	1470 / 2695 (54.5%)	1.08	4.16
JASPAR2022 CORE vertebrates non-redundant	MA0813.1	MA0813.1.TFAP2B	7.89e-4	9.66e-1	1.92e-3	300 / 2695 (11.1%)	230 / 2695 (8.5%)	1.30	2.88
uniprobe mouse	UP00016_1	Sry_primary	8.00e-4	9.79e-1	1.95e-3	1567 / 2695 (58.1%)	1451 / 2695 (53.8%)	1.08	5.56
JASPAR2022 CORE vertebrates non-redundant	MA1594.1	MA1594.1.ZNF382	8.78e-4	1.07e0	2.13e-3	137 / 2695 (5.1%)	90 / 2695 (3.3%)	1.52	4.93
JASPAR2022 CORE vertebrates non-redundant	MA1780.1	MA1780.1.TFAP4::FLI1	8.85e-4	1.08e0	2.14e-3	180 / 2695 (6.7%)	126 / 2695 (4.7%)	1.43	3.59
JASPAR2022 CORE vertebrates non-redundant	MA0163.1	MA0163.1.PLAG1	9.19e-4	1.12e0	2.21e-3	547 / 2695 (20.3%)	457 / 2695 (17.0%)	1.20	0.86
JASPAR2022 CORE vertebrates non-redundant	MA0865.2	MA0865.2.E2F8	9.28e-4	1.14e0	2.23e-3	1873 / 2695 (69.5%)	1765 / 2695 (65.5%)	1.06	5.64
JASPAR2022 CORE vertebrates non-redundant	MA0632.2	MA0632.2.TCFL5	9.54e-4	1.17e0	2.28e-3	241 / 2695 (8.9%)	179 / 2695 (6.6%)	1.34	11.38
JASPAR2022 CORE vertebrates non-redundant	MA1584.1	MA1584.1.ZIC5	9.81e-4	1.20e0	2.34e-3	190 / 2695 (7.1%)	135 / 2695 (5.0%)	1.40	4.98
JASPAR2022 CORE vertebrates non-redundant	MA1796.1	MA1796.1.ERF::FIGLA	9.83e-4	1.20e0	2.34e-3	621 / 2695 (23.0%)	527 / 2695 (19.6%)	1.18	2.31
JASPAR2022 CORE vertebrates non-redundant	MA0620.3	MA0620.3.MITF	1.02e-3	1.25e0	2.42e-3	702 / 2695 (26.0%)	604 / 2695 (22.4%)	1.16	6.65
JASPAR2022 CORE vertebrates non-redundant	MA0622.1	MA0622.1.Mlxip	1.02e-3	1.25e0	2.42e-3	213 / 2695 (7.9%)	155 / 2695 (5.8%)	1.37	12.23
JASPAR2022 CORE vertebrates non-redundant	MA0825.1	MA0825.1.MNT	1.13e-3	1.38e0	2.65e-3	86 / 2695 (3.2%)	50 / 2695 (1.9%)	1.71	13.52
JASPAR2022 CORE vertebrates non-redundant	MA1491.2	MA1491.2.GLI3	1.16e-3	1.42e0	2.73e-3	203 / 2695 (7.5%)	147 / 2695 (5.5%)	1.38	0.05
JASPAR2022 CORE vertebrates non-redundant	MA0908.1	MA0908.1.HOXD11	1.21e-3	1.48e0	2.84e-3	2644 / 2695 (98.1%)	2608 / 2695 (96.8%)	1.01	1.30
JASPAR2022 CORE vertebrates non-redundant	MA1538.1	MA1538.1.NR2F1	1.21e-3	1.49e0	2.84e-3	674 / 2695 (25.0%)	579 / 2695 (21.5%)	1.16	2.75
uniprobe mouse	UP00090_1	Elf3_primary	1.22e-3	1.49e0	2.84e-3	1314 / 2695 (48.8%)	1202 / 2695 (44.6%)	1.09	8.05
JASPAR2022 CORE vertebrates non-redundant	MA0481.3	MA0481.3.FOXP1	1.25e-3	1.53e0	2.91e-3	2455 / 2695 (91.1%)	2387 / 2695 (88.6%)	1.03	5.34
JASPAR2022 CORE vertebrates non-redundant	MA0042.2	MA0042.2.FOXI1	1.28e-3	1.56e0	2.96e-3	2417 / 2695 (89.7%)	2345 / 2695 (87.0%)	1.03	4.97
JASPAR2022 CORE vertebrates non-redundant	MA0625.2	MA0625.2.NFATC3	1.28e-3	1.57e0	2.96e-3	1108 / 2695 (41.1%)	999 / 2695 (37.1%)	1.11	8.90
JASPAR2022 CORE vertebrates non-redundant	MA0660.1	MA0660.1.MEF2B	1.42e-3	1.74e0	3.26e-3	1016 / 2695 (37.7%)	910 / 2695 (33.8%)	1.12	0.32
uniprobe mouse	UP00169_1	Lmx1b_3433.2	1.43e-3	1.75e0	3.27e-3	1019 / 2695 (37.8%)	913 / 2695 (33.9%)	1.12	6.89
JASPAR2022 CORE vertebrates non-redundant	MA2031.1	MA2031.1.SMAD2	1.46e-3	1.78e0	3.33e-3	1075 / 2695 (39.9%)	968 / 2695 (35.9%)	1.11	7.51
JASPAR2022 CORE vertebrates non-redundant	MA1531.1	MA1531.1.NR1D1	1.48e-3	1.81e0	3.37e-3	596 / 2695 (22.1%)	507 / 2695 (18.8%)	1.18	0.59
JASPAR2022 CORE vertebrates non-redundant	MA0517.1	MA0517.1.STAT1::STAT2	1.49e-3	1.82e0	3.38e-3	923 / 2695 (34.2%)	820 / 2695 (30.4%)	1.13	7.04
JASPAR2022 CORE vertebrates non-redundant	MA0144.2	MA0144.2.STAT3	1.49e-3	1.83e0	3.38e-3	2592 / 2695 (96.2%)	2545 / 2695 (94.4%)	1.02	1.92
uniprobe mouse	UP00168_1	Hoxd8_2644.1	1.53e-3	1.87e0	3.45e-3	2092 / 2695 (77.6%)	1998 / 2695 (74.1%)	1.05	5.20
JASPAR2022 CORE vertebrates non-redundant	MA1865.1	MA1865.1.ZNF574	1.54e-3	1.88e0	3.46e-3	1105 / 2695 (41.0%)	998 / 2695 (37.0%)	1.11	4.00
JASPAR2022 CORE vertebrates non-redundant	MA0683.1	MA0683.1.POU4F2	1.58e-3	1.93e0	3.54e-3	1068 / 2695 (39.6%)	962 / 2695 (35.7%)	1.11	2.39
JASPAR2022 CORE vertebrates non-redundant	MA0871.2	MA0871.2.TFEC	1.68e-3	2.06e0	3.77e-3	487 / 2695 (18.1%)	406 / 2695 (15.1%)	1.20	5.98
uniprobe mouse	UP00085_1	Sfpi1_primary	1.75e-3	2.15e0	3.92e-3	70 / 2695 (2.6%)	39 / 2695 (1.4%)	1.77	13.45
JASPAR2022 CORE vertebrates non-redundant	MA1802.1	MA1802.1.FOXO1::ELF1	1.76e-3	2.15e0	3.92e-3	988 / 2695 (36.7%)	885 / 2695 (32.8%)	1.12	7.50
JASPAR2022 CORE vertebrates non-redundant	MA0667.1	MA0667.1.MYF6	1.92e-3	2.35e0	4.26e-3	348 / 2695 (12.9%)	279 / 2695 (10.4%)	1.25	9.02
uniprobe mouse	UP00070_2	Gcm1_secondary	1.97e-3	2.41e0	4.36e-3	571 / 2695 (21.2%)	486 / 2695 (18.0%)	1.17	8.52
JASPAR2022 CORE vertebrates non-redundant	MA1568.1	MA1568.1.TCF21	2.06e-3	2.52e0	4.55e-3	1610 / 2695 (59.7%)	1505 / 2695 (55.8%)	1.07	4.88
JASPAR2022 CORE vertebrates non-redundant	MA1784.1	MA1784.1.ERF::FOXO1	2.07e-3	2.53e0	4.55e-3	1123 / 2695 (41.7%)	1019 / 2695 (37.8%)	1.10	7.07
uniprobe mouse	UP00029_1	Tbp_primary	2.16e-3	2.65e0	4.75e-3	727 / 2695 (27.0%)	635 / 2695 (23.6%)	1.14	8.69
uniprobe mouse	UP00061_1	Foxl1_primary	2.17e-3	2.66e0	4.75e-3	213 / 2695 (7.9%)	159 / 2695 (5.9%)	1.34	11.35
JASPAR2022 CORE vertebrates non-redundant	MA0788.1	MA0788.1.POU3F3	2.43e-3	2.98e0	5.31e-3	2545 / 2695 (94.4%)	2493 / 2695 (92.5%)	1.02	3.74
JASPAR2022 CORE vertebrates non-redundant	MA1537.1	MA1537.1.NR2F1	2.49e-3	3.04e0	5.41e-3	842 / 2695 (31.2%)	747 / 2695 (27.7%)	1.13	0.13
uniprobe mouse	UP00055_2	Hbp1_secondary	2.55e-3	3.12e0	5.53e-3	2644 / 2695 (98.1%)	2611 / 2695 (96.9%)	1.01	4.48
JASPAR2022 CORE vertebrates non-redundant	MA1621.1	MA1621.1.Rbpjl	2.58e-3	3.16e0	5.58e-3	2528 / 2695 (93.8%)	2474 / 2695 (91.8%)	1.02	2.69
JASPAR2022 CORE vertebrates non-redundant	MA1099.2	MA1099.2.HES1	2.78e-3	3.40e0	5.99e-3	1232 / 2695 (45.7%)	1130 / 2695 (41.9%)	1.09	5.46
JASPAR2022 CORE vertebrates non-redundant	MA0687.1	MA0687.1.SPIC	2.79e-3	3.42e0	6.00e-3	1645 / 2695 (61.0%)	1544 / 2695 (57.3%)	1.07	6.24
JASPAR2022 CORE vertebrates non-redundant	MA0822.1	MA0822.1.HES7	2.81e-3	3.44e0	6.02e-3	149 / 2695 (5.5%)	105 / 2695 (3.9%)	1.42	3.16
JASPAR2022 CORE vertebrates non-redundant	MA0259.1	MA0259.1.ARNT::HIF1A	3.07e-3	3.76e0	6.57e-3	297 / 2695 (11.0%)	236 / 2695 (8.8%)	1.26	11.56
JASPAR2022 CORE vertebrates non-redundant	MA1135.1	MA1135.1.FOSB::JUNB	3.12e-3	3.82e0	6.65e-3	60 / 2695 (2.2%)	33 / 2695 (1.2%)	1.79	14.17
JASPAR2022 CORE vertebrates non-redundant	MA1123.2	MA1123.2.TWIST1	3.26e-3	3.98e0	6.92e-3	980 / 2695 (36.4%)	884 / 2695 (32.8%)	1.11	8.01

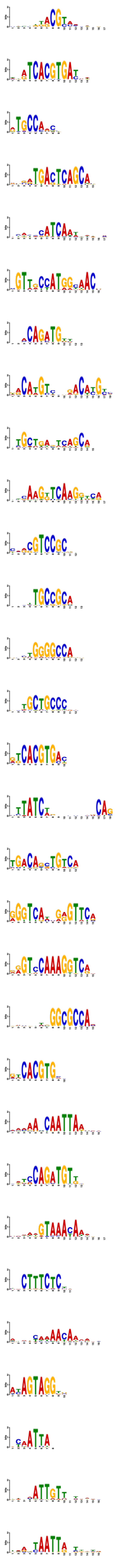

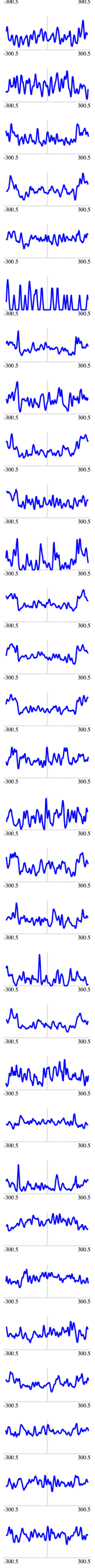

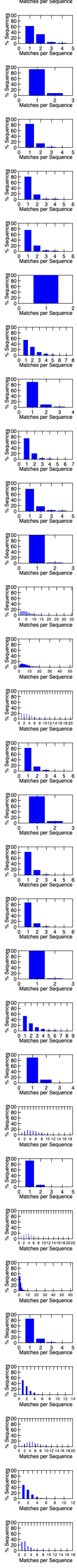

JASPAR2022 CORE vertebrates non-redundant	MA0615.1	MA0615.1.Gmeb1	3.27e-3	4.00e0	6.93e-3	291 / 2695 (10.8%)	231 / 2695 (8.6%)	1.26	10.85
JASPAR2022 CORE vertebrates non-redundant	MA0828.2	MA0828.2.SREBF2	3.38e-3	4.13e0	7.14e-3	283 / 2695 (10.5%)	224 / 2695 (8.3%)	1.26	2.80
JASPAR2022 CORE vertebrates non-redundant	MA0739.1	MA0739.1.Hic1	3.39e-3	4.15e0	7.14e-3	608 / 2695 (22.6%)	526 / 2695 (19.5%)	1.16	9.77
JASPAR2022 CORE vertebrates non-redundant	MA0591.1	MA0591.1.Bach1::Mafk	3.40e-3	4.16e0	7.15e-3	767 / 2695 (28.5%)	678 / 2695 (25.2%)	1.13	1.32
uniprobe mouse	UP00185_1	Pbx1_3203.1	3.41e-3	4.17e0	7.15e-3	1003 / 2695 (37.2%)	907 / 2695 (33.7%)	1.11	8.55
JASPAR2022 CORE vertebrates non-redundant	MA0600.2	MA0600.2.RFX2	3.42e-3	4.18e0	7.15e-3	24 / 2695 (0.9%)	8 / 2695 (0.3%)	2.78	12.88
JASPAR2022 CORE vertebrates non-redundant	MA1618.1	MA1618.1.Ptf1a	3.49e-3	4.28e0	7.29e-3	1383 / 2695 (51.3%)	1283 / 2695 (47.6%)	1.08	6.99
JASPAR2022 CORE vertebrates non-redundant	MA0861.1	MA0861.1.TP73	3.93e-3	4.81e0	8.17e-3	351 / 2695 (13.0%)	287 / 2695 (10.6%)	1.22	5.32
JASPAR2022 CORE vertebrates non-redundant	MA1520.1	MA1520.1.MAF	4.06e-3	4.96e0	8.41e-3	1004 / 2695 (37.3%)	910 / 2695 (33.8%)	1.10	6.53
JASPAR2022 CORE vertebrates non-redundant	MA1541.1	MA1541.1.NR6A1	4.15e-3	5.08e0	8.57e-3	628 / 2695 (23.3%)	547 / 2695 (20.3%)	1.15	7.14
JASPAR2022 CORE vertebrates non-redundant	MA0131.2	MA0131.2.HINFP	4.24e-3	5.19e0	8.75e-3	110 / 2695 (4.1%)	74 / 2695 (2.7%)	1.48	12.67
JASPAR2022 CORE vertebrates non-redundant	MA1583.1	MA1583.1.ZFP57	4.37e-3	5.35e0	8.96e-3	2524 / 2695 (93.7%)	2473 / 2695 (91.8%)	1.02	0.87
JASPAR2022 CORE vertebrates non-redundant	MA1615.1	MA1615.1.Plagl1	4.37e-3	5.35e0	8.96e-3	2610 / 2695 (96.8%)	2572 / 2695 (95.4%)	1.01	1.06
JASPAR2022 CORE vertebrates non-redundant	MA1728.1	MA1728.1.ZNF549	4.65e-3	5.69e0	9.52e-3	2457 / 2695 (91.2%)	2399 / 2695 (89.0%)	1.02	3.42
JASPAR2022 CORE vertebrates non-redundant	MA0636.1	MA0636.1.BHLHE41	4.73e-3	5.79e0	9.64e-3	812 / 2695 (30.1%)	725 / 2695 (26.9%)	1.12	0.06
JASPAR2022 CORE vertebrates non-redundant	MA0140.2	MA0140.2.GATA1::TAL1	4.74e-3	5.80e0	9.64e-3	200 / 2695 (7.4%)	152 / 2695 (5.6%)	1.31	10.45
JASPAR2022 CORE vertebrates non-redundant	MA1571.1	MA1571.1.TGIF2LX	4.84e-3	5.92e0	9.82e-3	790 / 2695 (29.3%)	704 / 2695 (26.1%)	1.12	6.12
JASPAR2022 CORE vertebrates non-redundant	MA0074.1	MA0074.1.RXRA::VDR	5.21e-3	6.38e0	1.06e-2	815 / 2695 (30.2%)	729 / 2695 (27.1%)	1.12	0.06
JASPAR2022 CORE vertebrates non-redundant	MA1494.1	MA1494.1.HNF4A	5.23e-3	6.40e0	1.06e-2	135 / 2695 (5.0%)	96 / 2695 (3.6%)	1.40	6.38
JASPAR2022 CORE vertebrates non-redundant	MA1727.1	MA1727.1.ZNF417	5.63e-3	6.89e0	1.13e-2	1362 / 2695 (50.5%)	1268 / 2695 (47.1%)	1.07	2.57
JASPAR2022 CORE vertebrates non-redundant	MA1464.1	MA1464.1.ARNT2	5.75e-3	7.04e0	1.15e-2	269 / 2695 (10.0%)	215 / 2695 (8.0%)	1.25	9.54
uniprobe mouse	UP00145_1	Barhl2_3868.1	5.78e-3	7.07e0	1.16e-2	2633 / 2695 (97.7%)	2601 / 2695 (96.5%)	1.01	0.55
JASPAR2022 CORE vertebrates non-redundant	MA0698.1	MA0698.1.ZBTB18	6.51e-3	7.96e0	1.30e-2	257 / 2695 (9.5%)	205 / 2695 (7.6%)	1.25	9.37
uniprobe mouse	UP00073_1	Foxa2_primary	6.82e-3	8.35e0	1.36e-2	2602 / 2695 (96.5%)	2565 / 2695 (95.2%)	1.01	2.64
JASPAR2022 CORE vertebrates non-redundant	MA0508.3	MA0508.3.PRDM1	6.94e-3	8.49e0	1.38e-2	1695 / 2695 (62.9%)	1606 / 2695 (59.6%)	1.06	6.94
uniprobe mouse	UP00061_2	Foxl1_secondary	6.94e-3	8.50e0	1.38e-2	613 / 2695 (22.7%)	538 / 2695 (20.0%)	1.14	9.53
JASPAR2022 CORE vertebrates non-redundant	MA1585.1	MA1585.1.ZKSCAN1	7.06e-3	8.64e0	1.40e-2	1792 / 2695 (66.5%)	1705 / 2695 (63.3%)	1.05	1.51
JASPAR2022 CORE vertebrates non-redundant	MA0879.2	MA0879.2.DLX1	7.83e-3	9.58e0	1.54e-2	2681 / 2695 (99.5%)	2664 / 2695 (98.8%)	1.01	4.49
uniprobe mouse	UP00064_1	Sox18_primary	7.92e-3	9.70e0	1.56e-2	1859 / 2695 (69.0%)	1775 / 2695 (65.9%)	1.05	7.24
uniprobe mouse	UP00151_1	Barx2_3447.2	8.15e-3	9.98e0	1.60e-2	1610 / 2695 (59.7%)	1522 / 2695 (56.5%)	1.06	5.82

Input Files

Alphabet

Background source: built from the control (negative) sequences

Order: 2 (only order-0 shown)

Name	Bg.				Bg.	Name
Adenine	0.3174	A	~	T	0.3174	Thymine
Cytosine	0.1826	C	~	G	0.1826	Guanine

Sequences

Primary Sequences	Number	Control Sequences	Number
promoters.fa	2994	primary sequences shuffled conserving 3-mer frequencies	2994

Motifs

Database	Source	Motif Count
uniprobe mouse	db/MOUSE/uniprobe_mouse.meme	386
JASPAR2022 CORE vertebrates non-redundant	db/JASPAR/JASPAR2022_CORE_vertebrates_non-redundant.meme	838

Other Settings

**Strand Handling**	Both the given and reverse complement strands are processed.
**Objective Function**	Differential Enrichment
**Statistical Test**	Fisher Exact Test
**Sequence Shuffling**	Control sequences are primary sequences shuffled preserving 3-mer frequencies.
**Hold-out Set**	10% of the input sequences were randomly assigned to the hold-out set to improve *p*-value accuracy.
**Pseudocount**	0.1 times the background frequencies
**Significance threshold**	Motifs must have an enrichment *E*-value of 10 or smaller.
**Random Number Seed**	0

SEA version
5.4.1 (Release date: Sat Aug 21 19:23:23 2021 -0700)

Reference
Timothy L. Bailey and Charles E. Grant, "SEA: Simple Enrichment Analysis of motifs", *BioRxiv*, 2021.

Command line summary

```
sea --verbosity 1 --oc . --thresh 10.0 --align center --p promoters.fa --m db/MOUSE/uniprobe_mouse.meme --m db/JASPAR/JASPAR2022_CORE_vertebrates_non-redundant.meme
```
